# A reciprocal feedback of Myc and lncRNA MTSS1-AS contributes to extracellular acidity-promoted metastasis of pancreatic cancer

**DOI:** 10.7150/thno.49147

**Published:** 2020-08-08

**Authors:** Yuhang Hu, Fan Wang, Fengyu Xu, Kaifeng Fang, Zhi Fang, Xiaoming Shuai, Kailin Cai, Jinhuang Chen, Ping Hu, Ding Chen, Peng Xu, Chaojie Hu, Zhu Zeng, Jianxin Zhong, Wei Li, Jiang Tang, Mengqi Huang, Yong Zhao, Chunyou Wang, Gang Zhao

**Affiliations:** 1 Department of Emergency Surgery, Union Hospital, Tongji Medical College, Huazhong University of Science and Technology, Wuhan 430022, China.; 2 Department of Gastroenterology Surgery, Union Hospital, Tongji Medical College, Huazhong University of Science and Technology, Wuhan 430022, China.; 3 Department of Pancreatic Surgery, Union Hospital, Tongji Medical College, Huazhong University of Science and Technology, Wuhan 430022, China.

**Keywords:** Acidity, LncRNA, metastasis, MTSS1-AS, Myc

## Abstract

**Rationale:** Previous studies have reported on the role of extracellular acidity in the metastasis of numerous cancers. However, the involvement of long noncoding RNA (lncRNA) in the extracellular acidity-induced cancer metastasis of pancreatic cancer (PC) remains unclear.

**Methods:** Different expression levels of lncRNAs in PC cells under normal and acidic conditions were compared by RNA sequencing (RNA-seq). The effects of antisense lncRNA of metastasis suppressor 1 (MTSS1-AS) on acidic PC cells were assessed by gain- and loss-of-function experiments. Fluorescence *in situ* hybridization, RNA immunoprecipitation, RNA pull-down, Western blot, luciferase reporter, and Chromatin immunoprecipitation assays were employed to determine the regulatory mechanisms of MTSS1-AS in the acidity-induced metastasis of PC cells. The expression of MTSS1-AS and associated pathways were compared in PC samples and peritumoral normal tissues.

**Results:** RNA-seq demonstrated that MTSS1-AS was significantly downregulated in pancreatic cells cultured with the acidic medium. The overexpression of MTSS1-AS remarkably inhibited the acidity-promoted metastasis of PC cells by upregulating the expression of its sense gene metastasis suppressor 1 (MTSS1). Mechanistically, MTSS1-AS scaffolded the interaction between E3 ubiquitin-protein ligase STIP1 homology and U-box containing protein 1 (STUB1) and transcription regulator myeloid zinc finger 1 (MZF1), leading to ubiquitination-mediated degradation of MZF1. Further, MZF1 inhibited the expression of MTSS1 by binding to the MTSS1 promoter. Thus, the acidity-reduced MTSS1-AS facilitated the stability of MZF1 and its inhibitory effect on MTSS1 transcription, thereby promoting the metastasis of PC cells under acidic conditions. Moreover, MTSS1-AS was transcriptionally repressed by the binding of MYC proto-oncogene (Myc) with initiator (Inr) elements of the MTSS1-AS promoter. Meanwhile, MTSS1-AS mutually repressed the expression of Myc by impairing the MZF1-mediated transcription activation of Myc, thereby forming a negative feedback loop between MTSS1-AS and Myc in acidic PC cells. In accordance with the experimental results, MTSS1-AS and MTSS1 were downregulated in PC and correlated with poor overall survival.

**Conclusions:** The results implicated that a reciprocal feedback loop between Myc and MTSS1-AS contributed to the extracellular acidity-promoted metastasis of PC, and indicated that MTSS1-AS was a valuable biomarker and therapeutic target for PC.

## Introduction

Pancreatic cancer (PC) is among the most lethal types of cancer worldwide, with the lowest 5-year survival rate 1. Early metastasis and diffuse invasion are the key reasons for the intractable clinical challenge represented by PC 2. Therefore, the mechanism underlying the invasion and metastasis of PC needs to be urgently investigated 3. Extracellular acidity is a well-known hallmark of the tumor microenvironment which can be attributed to the “Warburg effect”. Such a metabolism reprogram results in the occurrence of glycolysis even in the presence of abundant oxygen supply 4, leading to increased glucose metabolism and lactic acid production. Moreover, the erratic and limited vasculature within solid tumors results in the accumulation of lactic acid and H^+^ ions 5. Various types of cancer, particularly bulky and/or those with limited blood flow, display an acidic extracellular pH compared with normal tissue (6.2-6.9 *vs.* 7.3-7.4) 6-8. Emerging evidence indicated that extracellular acidity significantly promoted cancer invasion and metastasis 9, 10. As a typical solid tumor, PC forms an acidic extracellular microenvironment 11, 12. Previous studies reported that an acid-sensing ion channel contributed to the acidic-induced epithelial-mesenchymal transition (EMT) of PC through activating the Ca^2+^/Ras homolog family member A pathway 13. Moreover, some subsequent studies indicated that miR-652 participated in the acidity-induced metastasis of PC by directly regulating the expression of zinc finger E-box binding homeobox 1, which was the key transcription factor for EMT 14. Another study showed that laminin subunit gamma 2 promoted the expression and activity of Na^+^/H^+^ exchanger 1, which exaggerated extracellular acidification to induce dynamic actin-dependent EMT and invasion in PC cells 15. Although preliminary investigations on the acidity-induced metastasis of PC were performed, more precise studies are still needed to explore novel targets for the therapeutic treatment of PC.

Long non-coding RNAs (lncRNAs; RNAs comprising >200 nucleotides) cannot encode proteins. Alterations in lncRNAs disrupt various cellular processes, including cell differentiation, splicing or decay of RNAs, cell cycle progression, and epigenetic regulation within cancer cells 16. Increasing evidence confirmed that lncRNAs were crucial in the invasion and metastasis of human PC. For instance, the lncRNA maternally expressed gene 3 was demonstrated to function as a suppressor in PC by regulating the phosphatidylinositol 3-kinase signalling pathway 17. Kim et al. indicated that HOX transcript antisense RNA promoted the transcription repression of growth differentiation factor 15 by the enhancer of zeste homolog 2 to exhibit pro-oncogenic activity in PC 18. LncRNA-PLACT1 was shown to sustain the activation of nuclear factor kappa B pathway through forming a positive feedback loop with the NFKB inhibitor alpha/E2F transcription factor 1 axis to foster the metastasis of PC 19. Another study demonstrated that THAP9 antisense RNA 1 promoted the progression of PC via sponging miR-484 and interacting with Yes-associated protein 20. Zhou et al. showed the aberrant expression of paired mRNA/lncRNA in acidosis-adapted colorectal cancer cells, indicating that deregulated lncRNA might be involved in the acidosis adaption-enhanced growth and metastasis of colorectal cancer cells 21. Another study showed that acidosis-induced ribosomal intergenic spacer lncRNA operated as a molecular switch of regulating the structure and function of the nucleolus 22. Therefore, whether lncRNAs were involved in the acidity-induced metastasis of PC needed further exploration.

In the present study, RNA sequencing (RNA-seq) was performed to analyse the expression of lncRNAs derived from PC cells cultured with normal (pH 7.4) or acidic (pH 6.8) medium. The results showed that lncRNA (RP11-532M24.1), an antisense RNA of metastasis suppressor 1 (MTSS1) designated as lncRNA-MTSS1-AS, significantly decreased in acidic PC cells. Studies demonstrated that the MTSS1 level reduced in numerous types of cancer and inhibited the migration and invasion of different cancer cells 23-25. Additionally, MTSS1 was downregulated in PC and inversely correlated with advanced disease, early metastasis, and reduced survival 26. Coincidently, this study revealed that the overexpression of MTSS1-AS suppressed the acidity-promoted migration and invasion of PC cells by upregulating the expression of MTSS1. Therefore, the potential mechanism by which MTSS1-AS regulated the expression of MTSS1 was further investigated. Also, the regulatory mechanism of reduction of MTSS1-AS under acidic conditions was explored. In addition, the correlation between clinicopathology and expression of the MTSS1-AS/MTSS1 pathway was analyzed in PC samples.

## Materials and Methods

### Clinical samples

The clinical samples were obtained from Pancreatic Disease Institute of Union Hospital, Wuhan, China. We randomly selected 132 both PC and paired normal peritumoral (NP) tissue samples from patients without chemotherapy or radiotherapy before operation. Patients were undergoing surgical treatment according to the National Comprehensive Cancer Network (NCCN) guideline, including pancreatectomy, choledochojejunostomy, gastroenterostomy, and I^125^ seed implantation. The tissues were obtained from surgical resection of patients or biopsy of the palliative surgery patients. Part of the excised tissue specimens were fixed in 10% buffered formalin solution and then embedded in paraffin, and part of the samples were immediately frozen at liquid nitrogen after surgical resection. PC diagnosis in these individuals was based on original histopathology findings by two pathologists. The study was conducted in accordance with the Declaration of Helsinki. All samples were collected with the written informed consent of the patients, and the study was approved by the local Research Ethics Committee at the Academic Medical Centre of Huazhong University of Science and Technology.

### RNA-seq

RNA-seq analysis was performed by Sangon Biotech (Shanghai, China). Briefly, BxPC-3 cells were cultured in normal (pH 7.4) and acidic media (pH 6.8) for 24 h. Total RNAs were extracted from the two samples using TRIzol reagent (Invitrogen, USA). Purity of these samples were tested using Thermo Nanodrop 2000 (Thermo Fisher Scientific, USA) and the concentration were quantified accurately using Qubit™ RNA BR Assay Kit (Invitrogen). Ribo-Zero™ rRNA Removal Kit (Illumina, USA) was used to remove rRNA of samples. Then the strand specific cDNA library was well prepared and was tested properly by Agilent 2100 Bioanalyzer System (Agilent Technologies, USA). RNA-seq was performed on the Illumina HiSeq 2500 platform and RNA-seq dataset was visualized using the Qubit 2.0 Fluorometer (Invitrogen). High-quality data reads were consistent with publicly available NONCODE database. Gene expressions were normalized to fragments per kilobase of exon model per million mapped reads (FPKM) by Expectation Maximization (RSEM).

### Cell culture

The human PC cell lines (AsPC-1, PANC-1, SW1990, and BxPC-3) were obtained from American Type Culture Collection (ATCC, USA), while human pancreatic duct epithelial (HPDE) cell line was purchased from BeNa Culture Collection (BNCC, Beijing, China). They were tested and authenticated for genotypes by DNA fingerprinting within 6 months. Cells were cultured in RPMI-1640 (Gibco, USA) containing 10% fetal bovine serum (Gibco, USA) and 100 U/mL penicillin and 100 mg/mL streptomycin at 37 °C under 5% CO_2_. Some specific experiments were carried out with cells in exponential growth, cultured in acidic (pH 6.8) or normal (pH 7.4) medium for 24 or 48 h. To achieve media acidification, 20 mM Tris-(hydeoxymethyl)-aminomethane and 2-(N-morpholino)-ethane-sulfonic acid were utilized 27. The pH of the media was measured by a high-precision pH meter. All cultures were monitored routinely and found to be free of contamination by mycoplasma or fungi.

### Chromatin-immunoprecipitation (ChIP)

ChIP analyses were performed using the EZ-ChIP™ Kit (Millipore, MA, USA) according to manufacturer's instructions. For each ChIP assay, 1 × 10^6^ cells were fixed in 1% formaldehyde for 10 min at 37 °C and washed twice using ice cold PBS containing protease inhibitors. Subsequently, cells were scraped into conical tube and lysed with SDS lysis buffer supplemented with protease inhibitors. The chromatin DNA was sonicated and sheared to lengths between 200 and 1000 bp. After centrifugation, the supernatant was transferred to a new tube and diluted with ChIP dilution buffer containing protease inhibitors. Protein A agarose beads were removed to the diluted cell supernatant and agitated for 1 h at 4 °C to reduce the nonspecific background. The supernatant fraction was collected, and mixed with anti-MZF1 antibody (Santa Cruz Biotechnology, Shanghai, China) or anti-Myc (Cell Signaling, Danvers, MA, USA) and incubated overnight at 4 °C with rotation. Normal mouse IgG was served as a control, and an anti-RNA pol II antibody (Millipore) was used as the positive control. Protein A agarose beads were added to the reaction mixture for 1 h at 4 °C with rotation to collect the antibody/histone complex. Supernatant contained unbound and non-specific DNA was removed after gentle centrifugation. Protein A agarose/antibody/histone complex was washed on a rotating platform with the low salt immune complex wash buffer, high salt immune complex wash buffer, LiCl immune complex wash buffer and TE buffer. Elution buffer was used to separate the complex from the antibody. DNA samples were recovered by phenol/chloroform extraction and ethanol precipitation, and then amplified by PCR and separated by 2% agarose gel electrophoresis for analysis. The ChIP-qPCR primer sequences are given in Table S1. All the ChIP assays were repeated independently three times.

### Fluorescence *in situ* hybridization (FISH)

FISH analysis was conducted using the FISH Tag™ RNA Multicolour (Invitrogen, USA) and MAXIscript® (Ambion, USA) kits. The probe synthesis, labelling and purification procedures were following manufacturer's instructions. Total RNA was extracted from the PC cells using TRIzol reagent (Invitrogen). RNAs were amplified by qRT-PCR using primers containing a T7 promoter sequence specific for antisense of MTSS1-AS. After purification by PCR Clean Up Kit (Beyotime, Shanghai, China), the amplified product was transcribed into indicated RNA *in vitro* with the effect of T7 RNA polymerase. To purify the amine-modified RNA immediately, binding buffer with isopropanol was added to the synthesis reaction and mixed well. After centrifuged and washed by wash buffer and elution buffer repeatedly, the purified RNA was contained in collection tube and precipitated by ethanol. The concentration of the RNA was adjusted with water to a final concentration of 0.2 μg/μL. Subsequently, amine-modified RNA was labeled with fluorescent dye and purified again. The probe identified MTSS1-AS was labeled with green fluorescence. After ethanol precipitation, the fluorescent dye-labeled probe was well prepared. Cells were grown on cover glass and fixed with formaldehyde/PBT for 25 min with gentle rocking. Then the sample was permeabilized using Triton X-100 for 20 min and washed using PBT/50% hybridization buffer. Labeled probe was hybridized overnight with cell samples at 55 °C in a humid environment. Stained results were observed by using LSM 5 Pascal Laser Scanning Microscope (Zeiss Germany, Oberkochen, Germany). Probe sequence is given in Table S2.

### RNA pull-down

Total RNA was extracted from the PC cells using TRIzol reagent (Invitrogen), and was amplified by qRT-PCR using primers containing a T7 promoter sequence specific for MTSS1-AS or antisense of MTSS1-AS. Amplified product was purified using PCR Clean Up Kit (Beyotime), and then transcribed into indicated RNA *in vitro* using the MAXIscript™ T7 Transcription Kit (Thermo Fisher Scientific, MA, USA). Briefly, DNA template was mixed with T7 RNA polymerase, rNTPs, and transcription buffer, and the reaction mixture was incubated for 10 min to 1 h at 37 °C. The newly synthesized RNA was labeled with biotinylated cytidine bisphosphate using Pierce™ RNA 3' End Desthiobiotinylation Kit (Thermo Fisher Scientific, MA, USA). Lysates of BxPC-3 and PANC-1 cells were prepared using standard lysis buffers in Pierce™ Magnetic RNA-Protein Pull-Down Kit (Thermo Fisher Scientific). Streptavidin magnetic beads were bound to labeled RNA in RNA capture buffer firstly, and were then mixed with protein lysate, 50% glycerol and protein-RNA binding buffer for 1 h at 4 °C with agitation. After washing and elution, the RNA-binding protein complexes were heated and eluted for 10 min in boiling water. Retrieved proteins were detected by Western blot analysis. The antisense RNA was used as the negative control.

### RNA immunoprecipitation (RIP)

RIP assays were performed using the Magna RIP™ RNA-Binding Protein Immunoprecipitation Kit (Millipore) according to the manufacturer's instructions. Firstly, cell lysates were prepared using cell lysis buffer containing protease inhibitors and RNase inhibitors. Subsequently, magnetic beads were pre-incubated with an anti-MZF1 antibody (Santa Cruz Biotechnology) or IgG for 30 min at room temperature and washed by RIP wash buffer. The lysates were then immunoprecipitated with the bead-bound antibodies at 4 °C overnight in RIP immunoprecipitation buffer included with 0.5 M EDTA and 5 μL RNase inhibitor. Magnetic beads were reserved and washed repeatedly after centrifugation. To purify the RNA bound with protein, proteinase K buffer was used to re-suspend magnetic beads. Supernatant was removed, and the immunoprecipitate was mixed with salt solution I, salt solution II, precipitate enhancer and absolute ethanol overnight at -80 °C. Finally, the samples were centrifuged at 14,000 rpm for 15 min at 4 °C, and washed with 80% ethanol. Pellets were re-suspended in 10 to 20 μL of RNase-free water for analysis by qRT-PCR.

### Co-immunoprecipitation (Co-IP)

Cell lysates were prepared using Cell lysis buffer for IP (Beyotime) and adjusted to a final concentration of 1 μg/μL. 5% of cell lysates were retained as input group for direct Western blot analysis. For Co-IP group, protein A agarose beads were removed to the diluted cell supernatant and agitated for one hour at 4 °C to reduce the nonspecific background. Subsequently, cellular extracts were mixed overnight at 4 °C on a rotating platform with control IgG (Cell Signaling Technology) or following primary antibodies (4 μg/mL): rabbit anti-MZF1 (Santa Cruz Biotechnology), rabbit anti-STUB1 (Santa Cruz Biotechnology, Shanghai, China). Protein A/G PLUS-Agarose (Santa Cruz Biotechnology) was then added and the mixture was incubated for a further 2 h at 4 °C with agitation. The agarose was then separated, rinsed with lysis buffer and equivalent volumes of each sample were assessed by Western blot analysis.

### Northern blot analysis

MTSS1-AS-specific probes for Northern blot analysis were generated using a DIG RNA Labeling Kit (Roche, Germany) according to the manufacturer's instructions. Total RNA was extracted from the PC cells using standard TRIzol methods, and amplified using specific primers of MTSS1-AS by qRT-PCR. To obtain DIG-labeled DNA probes, the prepared DNA template was denatured in boiling water for 10 min and then incubated with DIG-High prime overnight at 37 °C. Total RNA was extracted again, and proceeded electrophoresis in denatured agarose gel for 2 h with formaldehyde gel electrophoresis buffer. To test the integrity of RNA, band signals were visualized by ChemiDoc™ XRS Molecular Imager System (Bio-Rad, USA). The prepared RNA samples were then transferred to nylon membrane (Millipore, USA) overnight using saline sodium citrate buffer. Membrane was stored for 2 h at 80 °C to fix RNA. DIG-labeled DNA probes were denatured in boiling water for 5 min and then hybridized with RNA on the membrane overnight at 55 °C on a rotating platform. Saline sodium citrate buffer contained with 0.1% SDS was used to wash the membrane for 15 min at 55 °C twice. Probes/MTSS1-AS complex on the membrane was incubated and stabilized on a rotating platform with the blocking solution, anti-digoxigenin-AP solution, wash buffer, detection buffer, and CSPD. Anti-digoxigenin-AP was utilized for capture of complex and CSPD was used to visualize probes. Images were collected by ChemiDoc™ XRS Molecular Imager System (Bio-Rad).

### Immunohistochemistry (IHC)

Immunohistochemistry was conducted using a previously described method 28. PC and matched NP tissues were collected from PC patients. Briefly, paraffin sections were placed at 60 °C for 2 h, and washed successively in dimethylbenzene solution, ethanol and TBS. Citrate buffer was utilized for antigen retrieval, while hydrogen peroxide was used to block endogenous peroxidase. Anti-MTSS1 antibody (Santa Cruz Biotechnology, Shanghai, China) or anti-MZF1 antibody (Santa Cruz Biotechnology) was used to incubate with tissues overnight at 4 °C. HRP-labeled goat anti-mouse/rabbit IgG (H+L) (Cell Signaling Technology) was added on paraffin section for secondary antibody binding reaction. Diaminobenzidine was used for visualization, and haematoxylin was used for re-staining nucleus. Samples were washed again in dimethylbenzene solution and ethanol, and sealed with neutral balsam. Immunohistochemical staining results were examined under a light microscope. Each sample was given an intensity score from 0 to 3 (units of intensity), and the average intensity score of each sample was calculated for each patient.

### Tumor xenograft assay

Male BALB/c nude mice (aged 4 weeks; n = 6/group) were obtained from HFK Bio-Technology, Beijing, China. For metastatic models, stable BxPC-3 (1 × 10^6^ cells/mouse) lines were injected into nude mice through the tail vein. After 4 weeks, the lungs and liver of the mice were removed and stained with H&E for enumeration of metastases. No blinding approach was used during this study. The Animal Research Committee of the Academic Medical Center at Huazhong University of Science and Technology (Wuhan, China) approved all aspects of this study. Care and handling of the animals were in accordance with the guidelines for Institutional and Animal Care and Use Committees.

### Statistical analysis

SPSS 24.0 was used for data analysis. Data were presented as means ± standard deviation (SD). Student's *t*-tests were used to compare data between groups, while Pearson correlation analysis was used to evaluate the correlation between MTSS1-AS and MTSS1 mRNA levels in patient samples. The relationships between the expression of MTSS1-AS and clinical characteristics of PC patients were analysed by *x^2^* test. Kaplan-Meier analysis with log-rank tests were conducted to analyse survival of PC patients. Receiver operating characteristic (ROC) curve analysis was used to evaluate the ability of biomarkers to forecast the mortality risk of PC. Arithmetic mean was used to divide high group and low group of RNA expression. An estimate of variation was performed within each group of data. The data meet the assumptions of the tests, respectively. *P* < 0.05 was considered to indicate statistical significance.

## Results

### MTSS1-AS remarkably decreased in PC cells under acidic conditions

**Figure 1:**
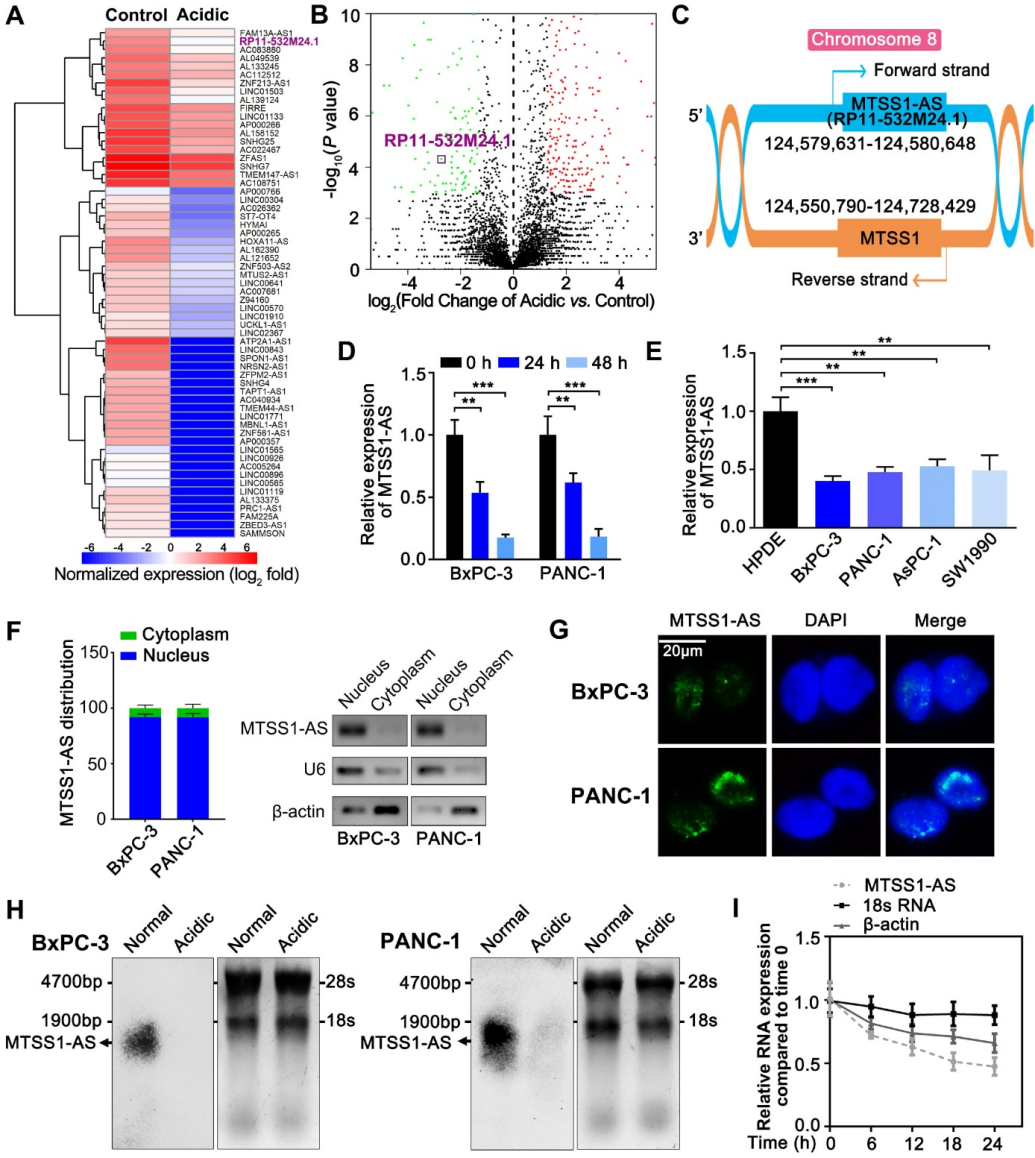
** MTSS1-AS is an acidity-downregulated lncRNA of PC cells. (A)** Heatmap of the differentially regulated lncRNAs from RNA-seq analysis of BxPC-3 cells cultured in normal or acidic. The expression levels of lncRNAs were normalized with log_2_ fold. Blue, low-expression; red, high-expression. The RP11-532M24.1 (MTSS1-AS) was marked with bold and purple. **(B)** Volcano plot showing the variations of lncRNA expression in acidic environment. We calculated the fold changes of expression of each lncRNA in acidic environment compared to normal environment from the results of RNA-seq, and normalized these fold changes with log_2_. Meanwhile, *P* values of these changes were -log_10_ normalized. Then, the log_2_ normalized values and -log_10_ normalized values of each lncRNA were plotted as the abscissa and the ordinate, respectively. Green spots, significant down-regulated lncRNAs in acidic environment; red spots, significant up-regulated lncRNAs in acidic environment. The RP11-532M24.1 (MTSS1-AS) was marked with purple box. **(C)** Genomic location of MTSS1-AS and MTSS1 with transcript orientation marked by arrows. **(D)** The qRT-PCR analysis of expression levels of MTSS1-AS in different BxPC-3/PANC-1 cells cultured in acidic (pH 6.8) medium for 0 h, 24 h and 48 h. **(E)** The expression levels of MTSS1-AS in different PC was compared with that of HPDE cells by qRT-PCR analysis.** (F)** Histogram showing qRT-PCR detected expression levels of MTSS1-AS in the subcellular fractions of BxPC-3/PANC-1 cells (Left). Then the qRT-PCR products were separated by 2% agarose gel electrophoresis; U6 and β-actin were used as markers of the nucleus and cytoplasm, respectively (Right). **(G)** FISH analysis of MTSS1-AS in BxPC-3/PANC-1 cells. The nucleus was counterstained with DAPI labelled by blue fluorescence. Green fluorescence represented MTSS1-AS. Scale bars: 20 µm. **(H)** The expression level of MTSS1-AS was verified by Northern blot analysis in BxPC-3/PANC-1 cells cultured in acidic medium (pH 6.8) or normal medium (pH 7.4). 18S rRNA and 28S rRNA were shown as references for MTSS1-AS length. **(I)** RNA synthesis in BxPC-3 cells was blocked by α-amanitin (50 µM), and MTSS1-AS stability was assessed by qRT-PCR compared with 0 h. RNA polymerase II and I were used to transcribe β-actin and 18S rRNA, respectively. All data were presented as means ± SD of at least three independent experiments. Values are significant at **P < 0.01 and ***P < 0.001 as indicated.

Although lncRNAs have been extensively researched in relation to tumorigenesis and progression, the changes in lncRNAs in the acidic microenvironment of cancer cells were unclear. In this study, the RNA-seq analysis was performed on the changes in the expression of lncRNA transcript in PC cells cultured in normal (pH 7.4) and acidic media (pH 6.8). The results revealed a prominently downregulated lncRNA, RP11-532M24.1 (Figure 1A-B). Considering the genomic location of RP11-532M24.1, lncRNA was identified as antisense MTSS1 and designated as MTSS1-AS (Figure 1C). As MTSS1 was a well-known anti-oncogene, MTSS1-AS was selected for further investigation. RNA-seq results were further verify using qRT-PCR to confirm that the expression levels of MTSS1-AS decreased in both BxPC-3 and PANC-1 cells during culture in an acidic medium (Figure 1D). In addition, the expression levels of MTSS1-AS were determined in the human pancreatic duct epithelial cell line (HPDE) and four PC cell lines (BxPC-3, PANC-1, AsPC-1 and SW1990). Reduced expression of MTSS1-AS was found in all PC cell lines (Figure 1E). The qRT-PCR analysis of the cytoplasmic and nuclear subcellular fractions showed that MTSS1-AS was located mainly in the nucleus (Figure 1F), which was further validated using FISH assay (Figure 1G). In addition, Northern blot analysis confirmed the reduction of MTSS1-AS in acidic PC cells using a specific RNA probe (Figure 1H). After blocking RNA synthesis in BxPC-3 cells via the RNA polymerase II inhibitor α-amanitin (50 μM), the expression MTSS1-AS was analyzed by qRT-PCR relative to 0 h. α-Amanitin treatment led to a marked decrease in the expression of MTSS1-AS, indicating that MTSS1-AS synthesis was mediated by RNA polymerase II. In contrast, α-amanitin did not affect the level of 18S mRNA, which was transcribed by RNA polymerase I (Figure 1I).

The full length MTSS1-AS sequence and its secondary structure were predicted according to minimum free energy using the NONCODE database 29 (Figure S1A-B). Predictions from online databases, including NONCODE, Coding Potential Assessment Tool 30, and Coding Potential Calculator 31, further supported the noncoding potential of MTSS1-AS (Figure S1C-E).

### MTSS1-AS apparently inhibited the migration and invasion of PC cells with or without acidic conditions

**Figure 2:**
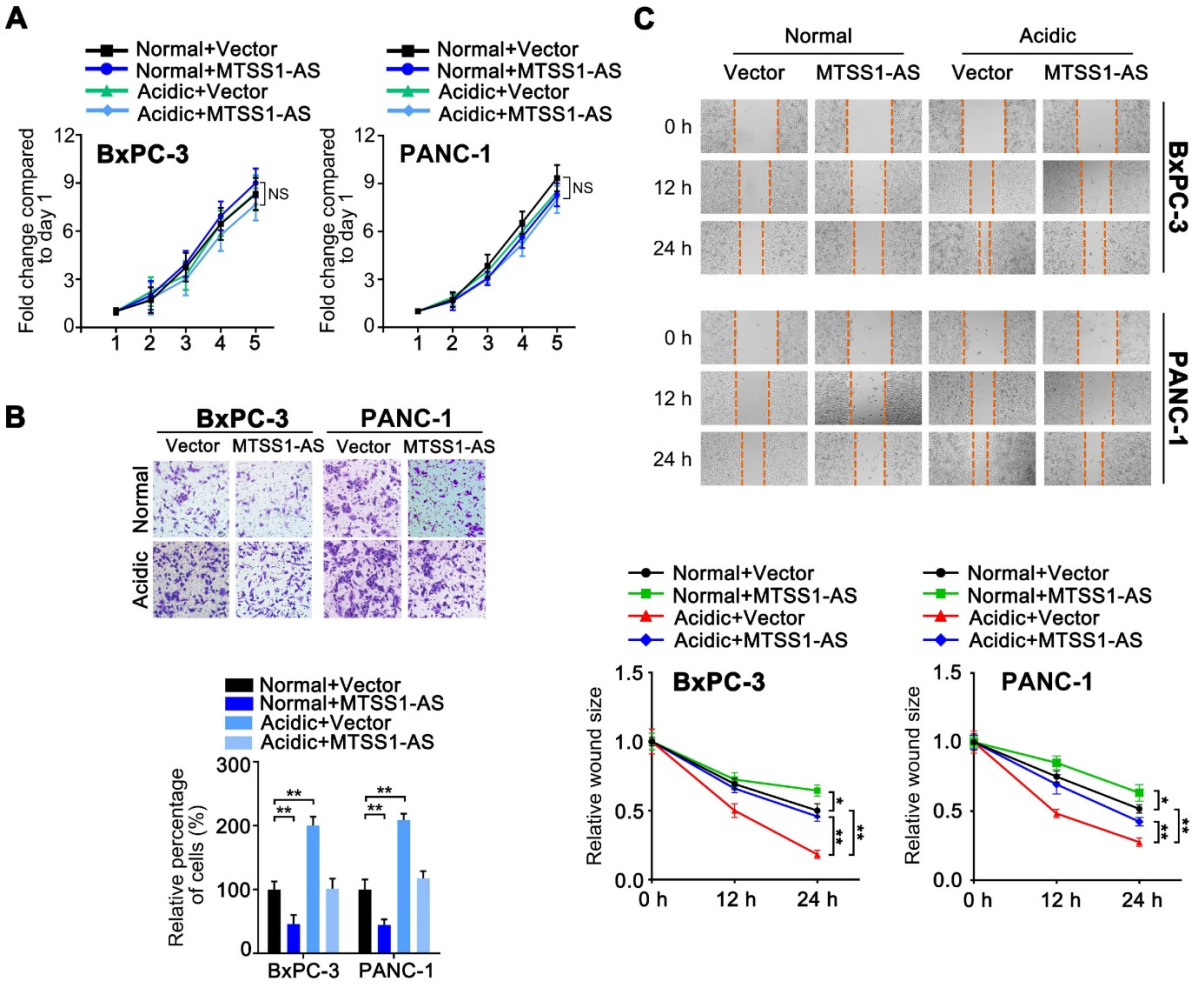
** MTSS1-AS apparently inhibited the migration and invasion of PC cells with or without acidic conditions. (A)** The BxPC-3/PANC-1 cells were transfected with vector plasmid (Vector) or MTSS1-AS overexpression plasmid (MTSS1-AS), and further cultured with normal medium or acidic medium, respectively. The proliferation of those cells was assessed by MTT assay over 5 days of culture. **(B)** The Transwell assay was performed to evaluate the invasion ability in the four groups of BxPC-3/PANC-1 cells. Representative images of invaded PC cells stained with crystal violet (Upper); histogram showing the relative percentage of invaded cells in the four groups (Below). **(C)** Wound-healing assay was performed to evaluate the migration ability in the four groups of BxPC-3/PANC-1 cells. Representative images of three time points (0 h, 12 h, 24 h) after would scratching of PC cells (Upper); line charts showing the relative wound size of three time points (0 h, 12 h, 24 h) in the four groups (Below). All data were presented as means ± SD of at least three independent experiments. Values are significant at *P < 0.05, **P < 0.01 as indicated. NS means the difference is not significant.

As a means of characterizing the role of MTSS1-AS in PC progression, an overexpression plasmid containing the MTSS1-AS sequence was used to upregulate the expression of MTSS1-AS in BxPC-3/PANC-1 cells cultured in normal or acidic medium. However, the overexpression of MTSS1-AS did not affect PC cell proliferation (Figure 2A). In contrast, the invasion and migration abilities of BxPC-3/PANC-1 cells significantly reduced after MTSS1-AS upregulation in both normal and acidic environments (Figure 2B-C). In addition, three short interference RNAs (siRNAs) specific for human MTSS1-AS were used to knock down the expression of MTSS1-AS in PC cells cultured in normal medium (Figure S2A). Two of the MTSS1-AS siRNAs, which significantly reduced the expression of MTSS1-AS, were used in downstream studies. MTSS1-AS knockdown had no significant impact on PC cell proliferation (Figure S2B). Matrigel-coated Transwell and wound-healing assays showed that the invasion and migration abilities of BxPC-3/PANC-1 cells transfected with siMTSS1-AS significantly increased (Figure S2C-D), suggesting that MTSS1-AS was involved in regulating the migration and invasion abilities of PC cells in acidic environments.

### MTSS1 was a critical target of MTSS1-AS exerting functions in PC cells under acidic conditions

**Figure 3:**
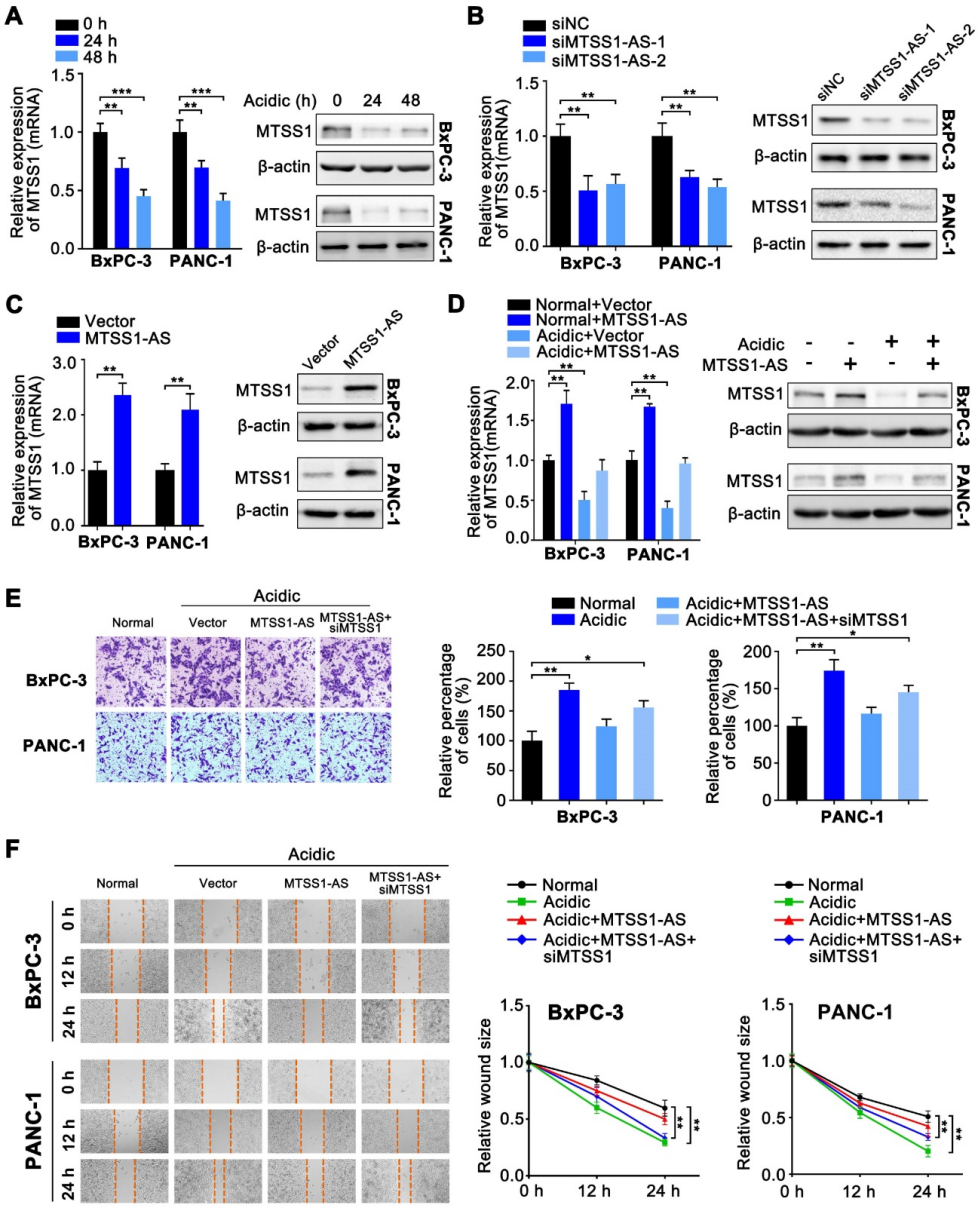
** MTSS1 is a critical target of MTSS1-AS exerting functions in PC cells during acidity. (A)** Expression of MTSS1 in BxPC-3/PANC-1 cells cultured with acidic medium for 0 h, 24 h and 48 h was analyzed at the mRNA and protein levels by qRT-PCR and Western blot analysis, respectively. **(B)** Expression of MTSS1 in BxPC-3/PANC-1 cells transfected with MTSS1-AS siRNAs (siMTSS1-AS-1, siMTSS1-AS-2) or the negative control siRNA (siNC) was analyzed at the mRNA and protein levels by qRT-PCR and Western blot analysis, respectively. **(C)** Expression of MTSS1 in BxPC-3/PANC-1 cells transfected with MTSS1-AS overexpression plasmid (MTSS1-AS) or the empty vector plasmid (Vector) was analyzed at the mRNA and protein levels by qRT-PCR and Western blot analysis, respectively. **(D)** MTSS1 in MTSS1-AS overexpressing-BxPC-3/PANC-1 cells cultured in normal or acidic environments was analyzed at the mRNA and protein levels by qRT-PCR and Western blot analysis, respectively. **(E-F)** The BxPC-3/PANC-1 cells were treated with four groups, which was transfected with vector plasmid (Vector), MTSS1-AS overexpression plasmid (MTSS1-AS) or co-transfected with MTSS1-AS and siMTSS1, and further cultured with normal medium or acidic medium, respectively.** (E)** Invasion ability of four groups of BxPC-3/PANC-1 cells were detected by the Transwell assay and representative images were shown (Left). Average counts from five randomly selected microscopic fields were analyzed. The histogram showed the percentage of invaded cells (Right). **(F)** Migration ability of four groups of BxPC-3/PANC-1 cells were measured in wound-healing assays. Representative images (Left) and relative wound size (Right) were shown. All data were presented as means ± SD of at least three independent experiments. Values are significant at *P < 0.05, **P < 0.01, ***P < 0.001 as indicated.

Since many studies shown that lncRNAs were involved in regulating their adjacent genes 32, it was hypothesized that MTSS1 was the functional target of MTSS1-AS. Coincidently, the expression of MTSS1 also decreased with the prolongation of culture in the acidic medium (pH 6.8) (Figure 3A). MTSS1-AS knockdown resulted in dramatically decreased MTSS1 in BxPC-3/PANC-1 cells at both the mRNA and protein levels (Figure 3B), while the overexpression of MTSS1-AS had the opposite effect (Figure 3C). Moreover, acidic treatment was found to reverse the increase in the mRNA and protein expression levels of MTSS1 induced by the overexpression of MTSS1-AS (Figure 3D), implicating MTSS1 as a direct target of MTSS1-AS under acidic conditions. Similarly, the invasion/migration of PC cells significantly decreased in MTSS1-AS-overexpressed cells under acidic conditions, and this effect was revised by siMTSS1 (Figure 3E-F). Thereby, the findings of this study suggested that MTSS1-AS played an inhibitory role in acidic PC cells by promoting MTSS1 transcription.

### MTSS1-AS relieved the inhibition of MZF1 on MTSS1 transcription

**Figure 4:**
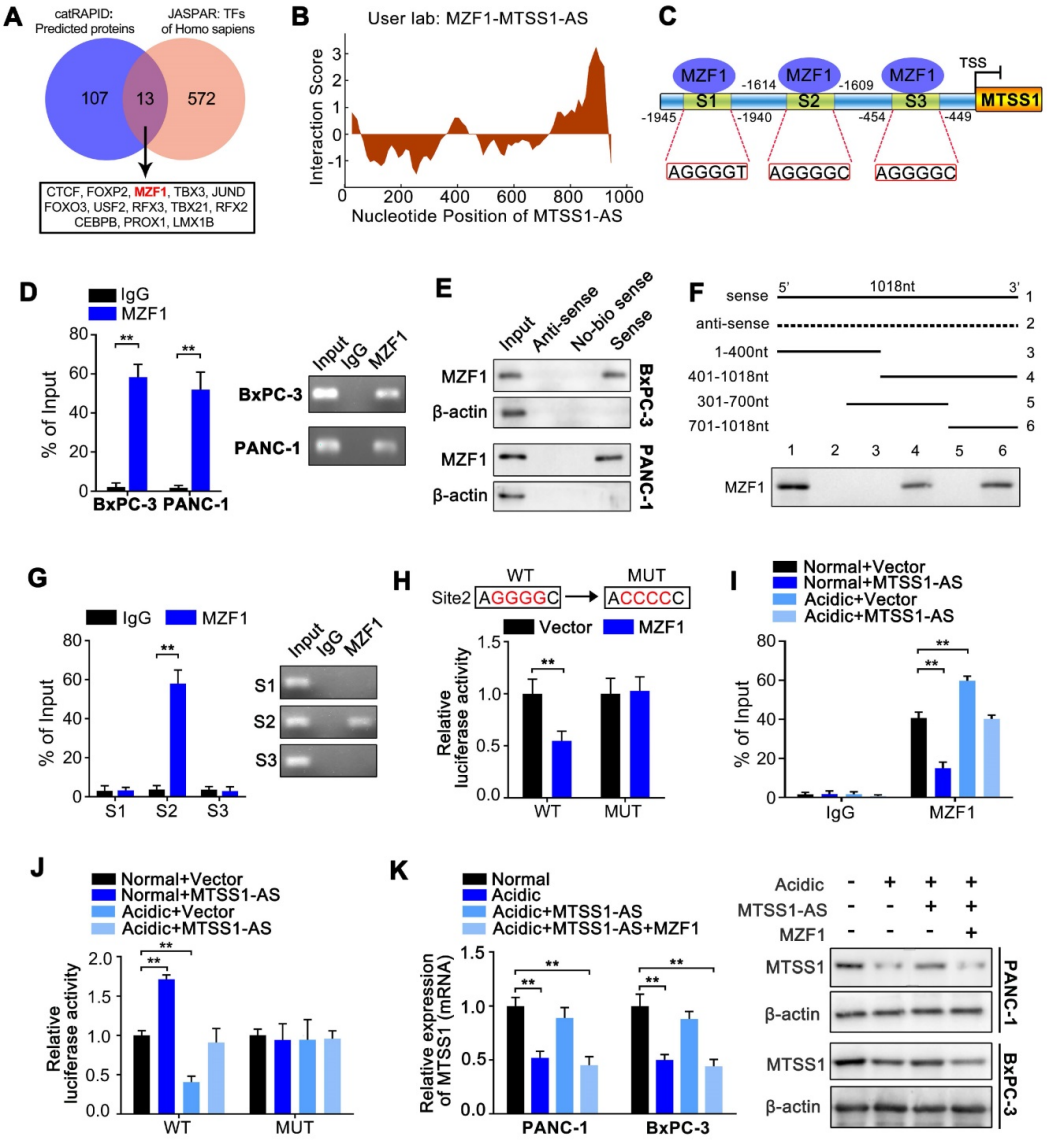
** MTSS1-AS relieved the inhibition of MZF1 on MTSS1 transcription. (A)** Venn diagram of the overlap in catRAPID (http://s.tartaglialab.com/page/catrapid_group) and JASPAR databases (http://jaspar.genereg.net) predictions indicating MZF1 as a MTSS1-AS/MTSS1-associated protein. The box below the arrow listed the 13 selected transcription factors. MZF1 was marked with bold and red. **(B)** RNA interaction profile from catRAPID suggesting that MTSS1-AS binds to the MZF1 protein. Predicted interaction score of MZF1 protein and nucleotide position of MTSS1-AS was shown via catRAPID. **(C)** Schematic illustration of the MTSS1 promoter region and the three putative MZF1 binding sites. The promoter sequence was 2000 base pairs of upstream of the transcription start site (TSS). **(D)** The RIP assays were used to probe BxPC-3/PANC-1 cell extracts, and qRT-PCR analysis was used to assess co-immunoprecipitated RNA (MTSS1-AS) levels following MZF1 or IgG (Left). Then the qRT-PCR products were separated by 2% agarose gel electrophoresis (Right). **(E)** Total BxPC-3/PANC-1 cell protein was utilized in a RNA pull-down experiment, with those proteins binding MTSS1-AS detected via Western blot analysis. Sense: transcribed and labeled MTSS1-AS; Anti-sense: transcribed and labeled antisense of MTSS1-AS; No-bio sense: transcribed but not labeled MTSS1-AS. **(F)** A series of MTSS1-AS deletion mutants were used for RNA pull-down experiments as above, followed by Western blot analysis with anti-MZF1. **(G)** The ChIP assays were used to assess the interaction between the MZF1 protein and the predicted binding site 2 of MTSS1 promoter in BxPC-3 cells (Left). The qRT-PCR products were separated by 2% agarose gel electrophoresis (Right).** (H)** The potential binding site 2 was mutated as indicated; WT: wild type, MUT: mutant type (Upper). Wild type or mutant type MTSS1 promoter activity was measured in luciferase reporter assays in BxPC-3 cells (Below).** (I)** After growth in normal or acidic media, BxPC-3 cells were transfected with vector or the MTSS1-AS overexpression plasmid. The binding between MZF1 and the MTSS1 promoter in transfected cells was detected by the ChIP assay. **(J)** WT or MUT MTSS1 promoter sequence luciferase reporter activity was assessed in BxPC-3 cells transfected with vector, MTSS1-AS or/and MZF1 overexpression plasmid under acidic conditions. **(K)** The expression levels of MTSS1 mRNA and protein were assessed by qRT-PCR and Western blot analysis, respectively, in BxPC-3/PANC-1 cells transfected with vector, MTSS1-AS or/and MZF1 overexpression plasmid and cultured under normal or acidic conditions. All data were presented as means ± SD of at least three independent experiments. Values are significant at **P < 0.01 as indicated. NS means the difference is not significant.

Next, the underlying mechanism by which MTSS1-AS regulated the expression of MTSS1 in PC cells was explored. Bioinformatics analyses was performed using the catRAPID 33 and JASPAR 34 databases to predict the probable underlying mechanism. The catRAPID database predicted possible interactions between MTSS1-AS and 120 proteins (Table S3). It was suspected that MTSS1-AS might interact with transcription factors to regulate transcription level of MTSS1. Coincidentally, 13 of the 120 proteins were known transcription factors of Homo species (Figure 4A). Furthermore, myeloid zinc finger 1 (MZF1) was shown to have the strongest binding ability to the MTSS1 promoter among the candidate transcription factors (Table S4). Thus, ultimately, the role of MZF1 in regulating MTSS1-AS/MTSS1 axis was emphasized. The catRAPID database suggested an interaction between MTSS1-AS and the transcription factor MZF1 (Figure 4B). Three binding sites between MZF1 and MTSS1 promoter were predicted using the JASPAR database (Figure 4C). MZF1 knockdown significantly increased the expression of MTSS1 at both the mRNA and protein levels in BxPC-3 cells, while the overexpression of MZF1 markedly reduced the expression of MTSS1 (Figure S3A-B). The expression of MTSS1 induced by the overexpression of MTSS1-AS was obviously reversed by the upregulation of MZF1 in both BxPC-3 and PANC-1 cells (Figure S3C). This result indicated that MZF1 was an inhibitor of the MTSS1-AS/MTSS1 pathway.

RIP assays using the anti-MZF1 antibody revealed distinct MTSS1-AS accumulation, while this effect was not observed in assays using the IgG control (Figure 4D). RNA pull-down assays using a MTSS1-AS probe showed that MZF1 was present in BxPC-3/PANC-1 extracts (Figure 4E). According to the prediction of the catRAPID database, the (5' 700-1018 nt 3') segment of MTSS1-AS had the highest probability of interaction with MZF1, and a probe depleted on this part of sequence failed to pull down the MZF1 protein (Figure 4F). This result suggested that the (5' 700-1018 nt 3') segment of MTSS1-AS was necessary for the binding between MZF1 and MTSS1-AS.

ChIP studies showed that MZF1 bound only to binding site 2 (S2) on the MTSS1 promoter (Figure 4G). Following mutation of binding site 2 from AGGGGC (wild type or WT) to ACCCCC (mutant type or MUT), the MTSS1 promoter activity was found to be repressed by the overexpression of MZF1 in WT BxPC-3 cells, while no significant change was observed in MUT BxPC-3 cells (Figure 4H). These results indicated that S2 was crucial to the mechanism by which MZF1 negatively regulated MTSS1 promoter activity. The ChIP assays also showed that the binding of MZF1 to the MTSS1 promoter increased under acidic conditions but decreased by overexpression of MTSS1-AS (Figure 4I). Similarly, the acidity-mediated inhibition of the transcriptional activity of the MTSS1 promoter was remarkably reversed by the overexpression of MTSS1-AS (Figure 4J). In addition, the overexpression of MTSS1-AS increased the acidity-mediated downregulation of the expression of MTSS1, which was reversed by the overexpression of MZF1 (Figure 4K). Similarly, the overexpression of MTSS1-AS decreased the migration and invasion abilities of PC cells cultured under acidic conditions, while these abilities were further enhanced by MZF1 upregulation (Figure S3D-E).

These results suggested that MZF1 was an inhibitor of MTSS1 transcription, while MTSS1-AS decreases the binding of MZF1 to the MTSS1 promoter. Therefore, acidity-mediated repression of MTSS1-AS promoted the binding between MZF1 and the MTSS1 promoter, thereby decreasing the expression of MTSS1 and promoting the metastasis of PC cells.

### MTSS1-AS destabilizes MZF1 protein through STUB1-dependent ubiquitination degradation

**Figure 5:**
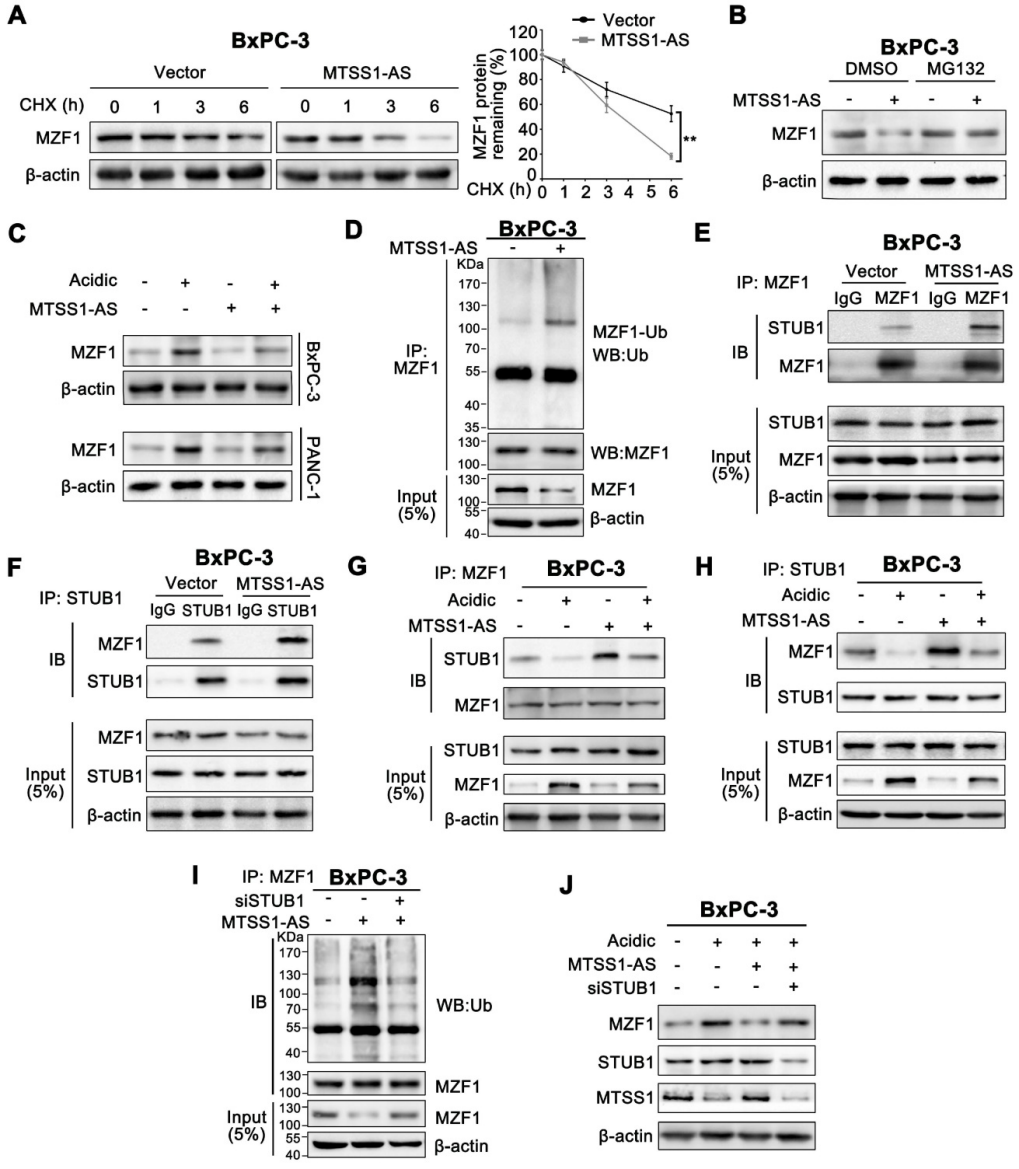
** MTSS1-AS destabilized MZF1 protein via STUB1-dependent ubiquitination degradation. (A)** BxPC-3 cells transfected using Vector or MTSS1-AS overexpression plasmid for 48 h, were exposed to 100 μg/mL cycloheximide (CHX) for the indicated periods of time. MZF1 protein levels were measured (Left). The line chart indicated relative quantification (Right). **(B)** BxPC-3 cells transfected with MTSS1-AS overexpression plasmid or vector were treated with MG132 (20 nM) or DMSO for 3 h before Western blot analysis for MZF1 protein levels.** (C)** MZF1 protein expression in BxPC-3/PANC-1 cells transfected with MTSS1-AS overexpression plasmid or vector and cultured in normal or acidic medium was measured by Western blot analysis. **(D)** Whole cell lysates of BxPC-3 cells transfected with the MTSS1-AS overexpression plasmid or vector were immunoprecipitated with MZF1 antibody. The ubiquitination of MZF1 was analyzed using anti-ubiquitin antibody in the cell lysates following anti-MZF1 immunoprecipitation. **(E-F)** After transfection with MTSS1-AS overexpression plasmid or vector, BxPC-3 cell lysates were immunoprecipitated with anti-MZF1 **(E)** or anti-STUB1 **(F)** antibody and then measured by Western blot analysis using anti-MZF1 and anti-STUB1, respectively. **(G-H)** Lysates of BxPC-3 cells transfected with the MTSS1-AS overexpression plasmid and cultured under acidic or normal conditions were immunoprecipitated with anti-MZF1 **(G)** or anti-STUB1 **(H)** and then measured by Western blot analysis using anti-MZF1 and anti-STUB1, respectively. **(I)** Lysates of BxPC-3 cells transfected with the MTSS1-AS overexpression plasmid or/and siSTUB1 were immunoprecipitated with anti-MZF1 before Western blot analysis using anti-MZF1 or anti-ubiquitin antibodies. **(J)** MZF1, STUB1 and MTSS1 proteins in BxPC-3 cells transfected with siSTUB1 and/or the MTSS1-AS overexpression plasmid and cultured under acidic or normal conditions were measured by Western blot analysis. All data were presented as means ± SD of at least three independent experiments. Values are significant at **P < 0.01 as indicated.

The relationship between MTSS1-AS and MZF1 was further investigated to fully understand the mechanism by which MTSS1-AS affected the interaction between MZF1 and the MTSS1 promoter. Figure S3C showed that the protein expression of MZF1 was obviously inhibited by the overexpression of MTSS1-AS, thus implicating that MZF1 was a direct target of MTSS1-AS. Nevertheless, in this study, the overexpression or knockdown of MTSS1-AS did not affect the expression of MZF1 at the mRNA level (Figure S4A-B). On the contrary, the protein expression of MZF1 significantly increased in MTSS1-AS-knockdown PC cells (Figure S4C). Following actinomycin D (Act-D) mediated inhibition of transcription, the qRT-PCR analysis showed that MZF1 mRNA half-life was unchanged in MTSS1-AS-overexpressing cells (Figure S4D). Therefore, these results indicated that MTSS1-AS did not affect the expression of MZF1 at the transcriptional level; instead, it acted at the posttranscriptional level. This was verified using the general protein translation inhibitor cycloheximide (CHX) to block protein synthesis. After CHX treatment, the stability of MZF1 significantly reduced by the overexpression of MTSS1-AS in PC cells (Figure 5A; Figure S4E). Furthermore, the proteasome inhibitor MG132 relieved the decrease in the MZF1 protein level in MTSS1-AS-overexpressing cells (Figure 5B; Figure S4F). In addition, the MZF1 protein level remarkably increased in BxPC-3/PANC-1 cells cultured under acidic conditions but was reduced by MTSS1-AS (Figure 5C). Consistently, MTSS1-AS upregulation in BxPC-3/PANC-1 cells led to markedly increased ubiquitination of MZF1, indicating that MTSS1-AS influenced MZF1 degradation via the ubiquitin-proteasome pathway (Figure 5D; Figure S4G).

Studies showed that STIP1 homology and U-box-containing protein 1 (STUB1) was responsible for the ubiquitination-mediated degradation of MZF1 35. Therefore, it was hypothesized that STUB1 was involved in the regulation of MTSS1-AS. Similarly, Co-IP assays revealed that STUB1 interacted with endogenous MZF1, and this interaction was effectively promoted by the overexpression of MTSS1-AS in BxPC-3/PANC-1 cells (Figure 5E-F; Figure S4H-I). Moreover, the combination of MZF1 and STUB1 was found to decrease in cells cultured in acidic medium, and this interaction was restored by the overexpression of MTSS1-AS (Figure 5G-H). Furthermore, the overexpression of MTSS1-AS obviously increased MZF1 ubiquitination, but was decreased by STUB1 knockdown (Figure 5I; Figure S4J). In accordance with this, the acidity-induced MZF1 upregulation was reversed by the overexpression of MTSS1-AS, which was rescued by STUB1 knockdown (Figure 5J; Figure S4K). Therefore, these results indicated that MTSS1-AS inhibited the protein expression of MZF1 by promoting STUB1-induced ubiquitination-mediated degradation of MZF1.

### Myc inhibited MTSS1-AS transcription of PC cells under acidic conditions

**Figure 6:**
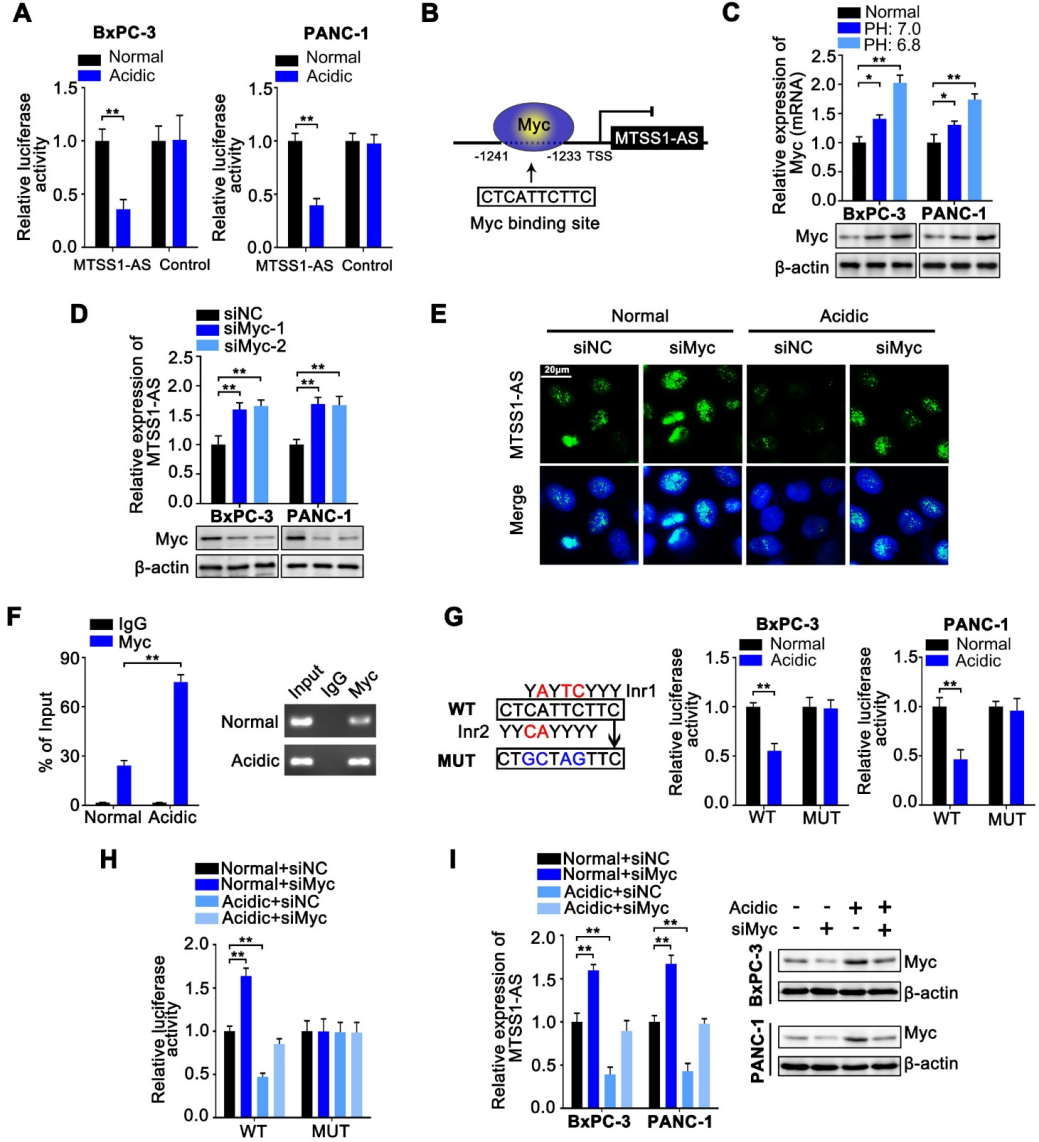
**Myc inhibited MTSS1-AS transcription of PC cells under acidic conditions. (A)** Luciferase activity of a reporter plasmid containing the MTSS1-AS promoter sequence or control pGL3-based vectors in BxPC-3/PANC-1 cells cultured under normal or acidic conditions was measured by luciferase reporter assay. Renilla luciferase served as a control. **(B)** Schematic illustration of MTSS1-AS promoter region and possible Myc binding sites. The promoter sequence was 2000 base pairs of upstream of the transcription start site (TSS). **(C)** Myc mRNA and protein levels in BxPC-3/PANC-1 cells cultured under different acidic conditions were assessed by qRT-PCR and Western blot analysis, respectively. **(D)** The expression of MTSS1-AS in Myc knockdown BxPC-3/PANC-1 cells were analyzed by qRT-PCR. Knockdown efficiency of two siRNA for Myc (siMyc-1, siMyc-2) were measured by Western blot analysis.** (E)** FISH assays were performed to detect MTSS1-AS in BxPC-3 cells transfected with siNC or siMyc under normal or acidic conditions. The nucleus was counterstained with DAPI labelled by blue fluorescence. Green fluorescence represented MTSS1-AS. Scale bars: 20 µm. **(F)** The ChIP assays were performed to identify the ability of Myc protein to bind the MTSS1-AS promoter in BxPC-3 cells cultured under normal or acidic conditions (Left). The qRT-PCR products were separated by 2% agarose gel electrophoresis (Right). **(G)** The potential binding site was mutated as indicated. The construction of two types of Inr elements was shown, compared with the predicted Myc binding site (Left). WT or MUT MTSS1-AS promoter sequence luciferase reporter activity in BxPC-3/PANC-1 cells cultured under normal or acidic conditions was measured by luciferase reporter assay. Renilla luciferase served as a control (Right). **(H)** WT or MUT MTSS1-AS promoter sequence luciferase reporter activity in BxPC-3 cells transfected with siNC or siMyc and cultured under normal or acidic conditions was assessed by luciferase reporter assay. **(I)** After BxPC-3/PANC-1 cells were transfected with siNC or siMyc and cultured under normal or acidic conditions, the expression of MTSS1-AS and Myc protein were assessed by qRT-PCR or Western blot analysis, respectively. All data were presented as means ± SD of at least three independent experiments. Values are significant at *P < 0.05, **P < 0.01 as indicated.

A luciferase reporter plasmid containing the promoter region of MTSS1-AS was transfected into BxPC-3 and PANC-1 cells to investigate the mechanisms by which the expression of MTSS1-AS was regulated in acidic microenvironments. In luciferase reporter assays, the luciferase activity obviously decreased in cells cultured in acidic medium, indicating that the transcriptional activity of MTSS1-AS was inhibited under acidic conditions (Figure 6A). MYC proto-oncogene (Myc) is a well-known multifunctional transcription factor often dysregulated in cancer and involved in metastasis, stress responses, and metabolism 36. Previous studies showed an upregulation of Myc in cells exposed to extracellular acidity 37. Myc has been shown to exert its inhibitory effect on downstream target genes by binding with the consensus transcription initiation motif in certain gene promoters 38, 39. Similarly, DNA sequence analysis showed that the MTSS1-AS promoter region contained a Myc-specific binding site (Figure 6B). In addition, the expression of Myc considerably increased under acidic conditions compared with normal conditions (Figure 6C). Therefore, it was speculated that MTSS1-AS was transcriptionally regulated by Myc. Furthermore, both qRT-PCR and FISH assays demonstrated that Myc depletion led to the notable upregulation of the expression of MTSS1-AS under both normal and acidic conditions (Figure 6D-E). The ChIP assays revealed binding between Myc and MTSS1-AS promoter, which increased under acidic conditions (Figure 6F). The regulation of MTSS1-AS by Myc was further verified by mutating the WT Myc to generate a MUT binding site in the MTSS1-AS promoter of BxPC-3 cells. Specific MTSS1-AS promoter sequences containing the WT or MUT were inserted into the promoter region of the luciferase reporter plasmid. The results showed that the MTSS1-AS promoter activity decreased in WT cells, but not in MUT cells cultured under acidic conditions (Figure 6G). The MTSS1-AS promoter luciferase assay also showed that Myc depletion increased MTSS1 promoter activity, which was inhibited during culture in acidic medium (Figure 6H). As expected, the expression of acidity-repressed MTSS1-AS was remarkably upregulated in Myc-depleted cells (Figure 6I). Consistently, Myc knockdown upregulated the expression of MTSS1 in both BxPC-3 and PANC-1 cells under acidic conditions, which was reversed by MTSS1-AS knockdown (Figure S5A). The Matrigel-coated Transwell and wound-healing assays showed that the invasion and migration abilities of BxPC-3/PANC-1 cells transfected with siMyc significantly decreased, while were recovered after co-transfection with siMyc and siMTSS1-AS (Figure S5B-C). Therefore, these results implied that the downregulation of MTSS1-AS under acidic culture conditions was transcriptionally regulated by Myc, and the Myc/MTSS1-AS axis was required for MTSS1 regulation.

### MTSS1-AS1 reciprocally inhibited the MZF1-dependent transcription of Myc

**Figure 7:**
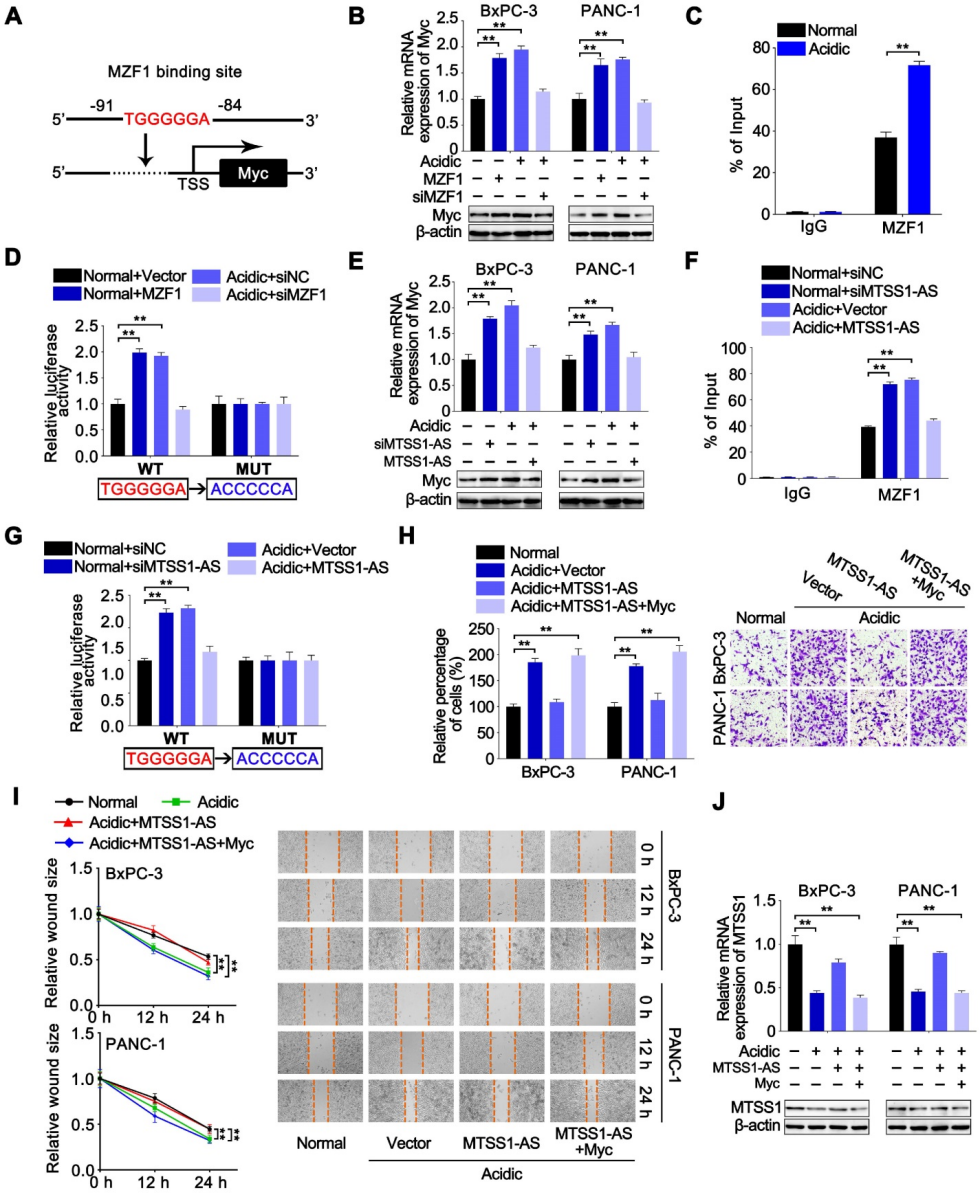
**MTSS1-AS1 reciprocally inhibited MZF1-dependent transcription of Myc. (A)** Schematic illustration of Myc promoter region and possible MZF1 binding sites. The promoter sequence was 2000 base pairs of upstream of the transcription start site (TSS). **(B)** Myc mRNA and protein levels were assessed by qRT-PCR and Western blot analysis respectively in BxPC-3/PANC-1 cells transfected with MZF1 overexpression plasmid or siMZF1 and cultured in normal or acidic medium. **(C)** The ChIP assays were performed to identify the ability of MZF1 protein to bind the Myc promoter in BxPC-3 cells cultured under normal or acidic conditions. **(D)** BxPC-3 cells were transfected with MZF1 overexpression plasmid or siMZF1 with respective negative control, and cultured under normal or acidic conditions. WT or MUT Myc promoter sequence luciferase reporter activity in prepared cells previously was assessed by luciferase reporter assay. The potential binding site was mutated as indicated; WT: wild type, MUT: mutant type. **(E)** Myc mRNA and protein levels were assessed by qRT-PCR and Western blot analysis respectively in BxPC-3/PANC-1 cells transfected with siMTSS1-AS or MTSS1-AS overexpression plasmid and cultured in normal or acidic medium. **(F-G)** BxPC-3 cells were transfected with siMTSS1-AS or MTSS1-AS overexpression plasmid and respective negative control, and cultured under normal or acidic conditions. **(F)** The ChIP assays were then performed to identify the ability of MZF1 protein to bind the Myc promoter in prepared cells previously. **(G)** WT or MUT Myc promoter sequence luciferase reporter activity in prepared cells previously was assessed by luciferase reporter assay. The potential binding site was mutated as indicated; WT: wild type, MUT: mutant type.** (H-I)** The BxPC-3/PANC-1 cells were treated with four groups, which was transfected with vector plasmid (Vector), MTSS1-AS overexpression plasmid (MTSS1-AS) or co-transfected with MTSS1-AS and Myc, and further cultured with normal medium or acidic medium, respectively.** (H)** Invasion ability of four groups of cells was detected by the Transwell assay, and the histogram shows the percentage of invaded cells (Left). Representative images of invaded PC cells were shown (Right). **(I)** Migration ability of four groups of BxPC-3/PANC-1 cells were measured in wound-healing assays. Relative wound size (Left) and representative images (Right) are shown. **(J)** MTSS1 mRNA and protein levels were assessed by qRT-PCR and Western blot analysis respectively. All data were presented as means ± SD of at least three independent experiments. Values are significant at **P < 0.01 as indicated.

Studies demonstrated that Myc transcription was upregulated by liver kinase B1 loss-mediated expression of MZF1, and the MZF1/Myc axis was responsible for migration and invasion in lung adenocarcinoma cells 40. Therefore, whether Myc could be regulated by the MTSS1-AS/MZF1 pathway needed further exploration. The JASPAR database showed that the Myc promoter region contained a potential binding site for MZF1 (Figure 7A). Indeed, the overexpression of MZF1 obviously promoted the expression of Myc under normal conditions, while MZF1 knockdown significantly inhibited the expression of Myc at both mRNA and protein levels in BxPC-3 and PANC-1 cells under acidic conditions (Figure 7B). Coincidently, the ChIP assays confirmed the binding between MZF1 and Myc promoter (Figure 7C). Furthermore, the binding of MZF1 on Myc promoter significantly increased in BxPC-3 cells under acidic conditions. Dual-luciferase reporter assay was conducted by transfection with a dual-luciferase reporter containing a WT or MUT binding site for MZF1 in the promoter region to validate the activity of the binding between MZF1 and Myc promoter. The results showed that the luciferase density increased in BxPC-3 cells transfected with WT reporter under acidic conditions and was restored by MZF1 knockdown, but no obvious alteration was observed in BxPC-3 cells transfected with the MUT reporter (Figure 7D). These data suggested that Myc transcription was also upregulated by MZF1 in PC cells. The MTSS1-AS knockdown obviously promoted the expression of Myc under normal conditions, while the overexpression of MTSS1-AS inhibited the expression of Myc in BxPC-3 and PANC-1 cells under acidic conditions (Figure 7E). The ChIP assays showed that the MTSS1-AS knockdown significantly increased the binding between MZF1 and Myc promoter. On the contrary, the overexpression of MTSS1-AS obviously inhibited the binding between MZF1 and Myc promoter, which was induced by acidic conditions in BxPC-3 cells (Figure 7F). Meanwhile, the increase in luciferase density induced by acidic condition was inhibited by transfection with MTSS1-AS vector (Figure 7G). The overexpression of MTSS1-AS decreased the acidity-induced invasion and migration abilities of BxPC-3/PANC-1 cells, while were recovered after co-transfection with MTSS1-AS and Myc (Figure 7H-I). Similarly, the mRNA and protein expression levels of MTSS1 in acidic PC cells also significantly increased by the overexpression of MTSS1-AS, which were reversed by the overexpression of Myc (Figure 7J). Together, these results implied that the MTSS1-AS/MZF1 pathway regulated the expression of Myc at the transcription level, and the Myc-induced downregulation of MTSS1-AS under acidic conditions further increased the expression of Myc to form a reciprocal feedback between Myc and MTSS1-AS.

### Acidity-induced Myc/MTSS1-AS signalling was involved in the metastasis of PC

**Figure 8:**
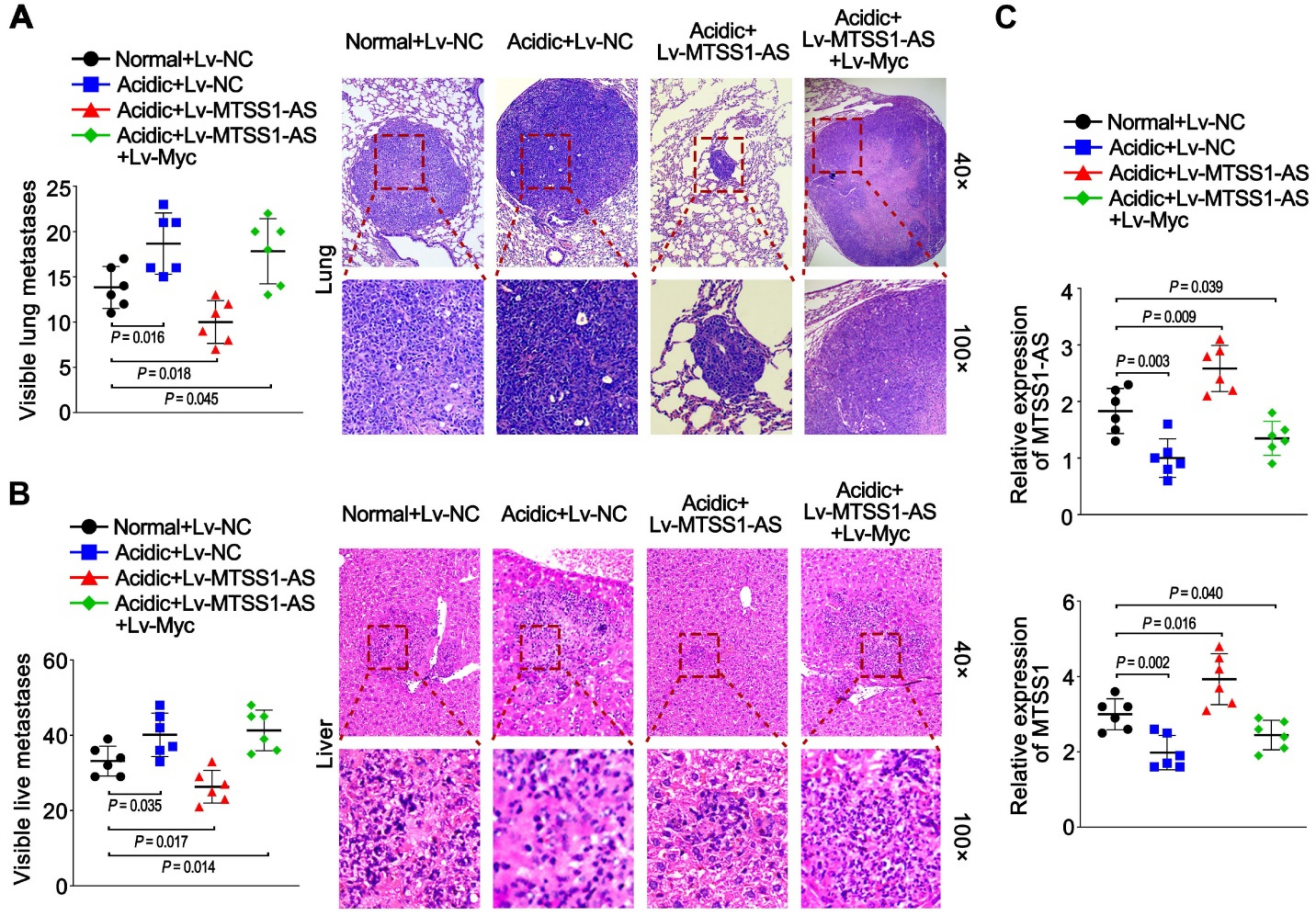
** Acidity-induced Myc/MTSS1-AS signalling was involved in metastasis of PC.** BxPC-3 cells were cultured for a long time under normal or acidic conditions. Nude mice were injected via the tail vein with those BxPC-3 cells stably expressing control vector (Lv-NC), MTSS1-AS (Lv-MTSS1-AS) or MTSS1-AS/Myc (Lv-MTSS1-AS + Lv-Myc) (1 × 10^6^ cells per mouse) to observe metastasis. **(A)** Histogram showing the number of visible lung metastases in five sections from each mouse (Left). Representative images of lungs and H&E stained sections (Right).** (B)** Histogram showing the number of visible liver metastases in five sections from each mouse (Left). Representative H&E stained sections of livers with metastases (Right).** (C)** MTSS1-AS and MTSS1 expression in lung metastatic tumor tissues were detected by qRT-PCR. All data were presented as means ± SD of at least three independent experiments.

BxPC-3 cells were cultured for a long time under normal or acidic conditions to further reveal the effects of the Myc/MTSS1-AS pathway on metastasis of PC *in vivo* and then stably transfected with lentivirus-containing negative control (Lv-NC), or MTSS1-AS (Lv-MTSS1-AS), or co-transfected with Lv-MTSS1-AS and lentivirus-containing Myc (Lv-Myc). Subsequently, these cells were injected into nude mice via the tail vein. Long-term acidic cultured PC cells caused more visible lung and liver metastases in mice compared with the normal group. Meanwhile, the mice in the Lv-MTSS1-AS group had decreased visible lung and liver metastases compared with the Lv-NC group when injected with PC cells cultured under acidic conditions for a long term, which were recovered in co-transfection with Lv-Myc group (Figure 8A-B). Coincidently, the expression of MTSS1-AS and MTSS1 in lung metastatic tumor tissues significantly increased in the Lv-MTSS1-AS group but inversely decreased after co-transfection with Lv-Myc (Figure 8C). These results suggest that the acidity-induced Myc/MTSS1-AS pathway was involved in the metastasis of PC.

### MTSS1-AS was downregulated in PC and correlated with poor clinical outcomes

**Figure 9:**
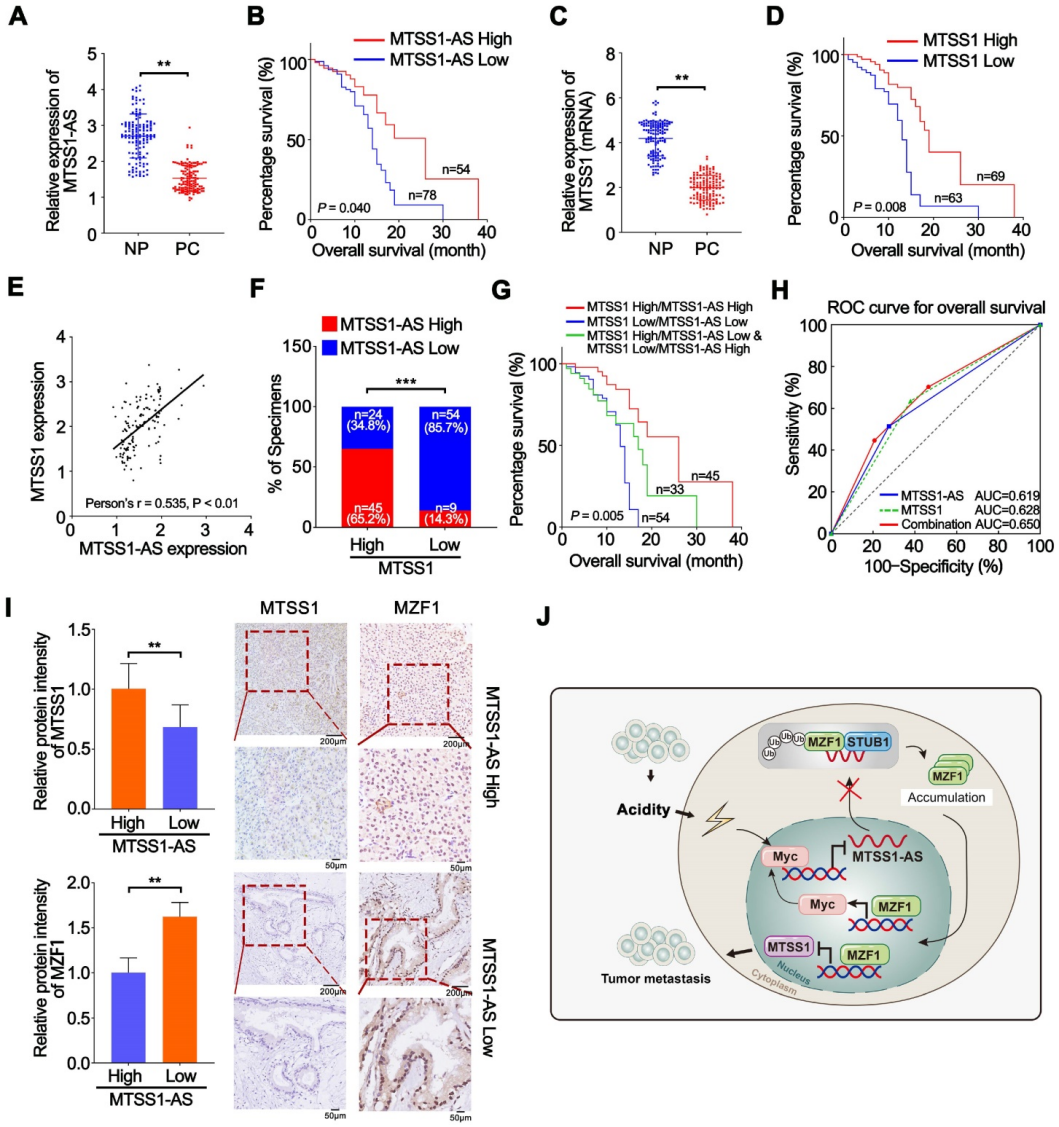
** MTSS1-AS was downregulated in PC and correlates with poor clinical outcome. (A)** The expression of MTSS1-AS was detected by qRT-PCR in 132 paired NP and PC tissues from patients with PC. **(B)** Overall survival of 132 patients with PC was analyzed by generation of Kaplan-Meier curves. The low or high MTSS1-AS groups are below or above the 50th percentile of MTSS1-AS expression, respectively. **(C)** The expression of MTSS1 was detected by qRT-PCR in 132 paired NP and PC tissues from patients with PC.** (D)** Overall survival of 132 patients with PC were analyzed by generation of Kaplan-Meier curves. The low or high MTSS1 groups are below or above the 50th percentile of MTSS1 expression, respectively. **(E)** Pearson's correlation analysis was used to verify the relationship of the expression between MTSS1-AS and MTSS1 in 132 patients with PC. **(F)** Correlation between MTSS1-AS and MTSS1 in specimens of patients with PC were analyzed by *chi*-square test.** (G)** Kaplan-Meier analysis of overall survival for patients with PC (n=132) based on the number of upregulated molecular markers including MTSS1-AS and MTSS1. Patients were divided into three groups based on the expression of MTSS1-AS and MTSS1 at RNA level as indicated. **(H)** ROC curve analysis for overall survival for MTSS1-AS [AUC=0.619, (95% CI, 0.523-0.715), *P*=0.019], MTSS1 [AUC=0.628, (95% CI, 0.532-0.724), *P*=0.012] as individual biomarkers or for the combined panel [AUC=0.650, (95% CI, 0.556-0.744), *P*=0.003]. Setting parameters were as follows: death as positive event; score of low MTSS1-AS or MTSS1 samples was 1, while high MTSS1-AS or MTSS1 samples was 0. Score of the combined panel was the sum of the scores of each sample. Area under the curve (AUC) was used to evaluate predictive capability. 95% CI, 95% confidence interval. **(I)** MTSS1 and MZF1 expression in NP and PC tissues from patients analyzed by IHC. Histogram showing the relative protein intensity via IHC scores (Left). Representative images of stained sections were indicated (Right). **(J)** Schematic diagram of the mechanism by which MTSS1-AS regulates PC in normal and acidic environments. Values are significant at **P < 0.01, ***P < 0.001 as indicated.

The expression of MTSS1-AS was detected via PCR analysis in 132 paired PC and NP tissues from patients with PC to further validate the relationship between the expression of MTSS1-AS and the progression of PC. MTSS1-AS transcript levels remarkably reduced in the PC tissues compared with those in paired noncancerous tissues (Figure 9A). Furthermore, the downregulation of MTSS1-AS in patients with PC significantly correlated with lymphatic invasion (*P* = 0.0270), vascular infiltration (*P* = 0.0298), and distant metastasis (*P* = 0.0108) (Table S5). The Kaplan-Meier survival analysis showed that patients with PC having low MTSS1-AS levels were associated with poor overall survival (Figure 9B). Meanwhile, lower MTSS1 levels were also observed in PC tissues compared with paired noncancerous tissues (Figure 9C) and correlated with shorter overall survival (Figure 9D). Moreover, the expression of MTSS1-AS and MTSS1 showed a positive correlation in patients with PC, as detected by Pearson's correlation analysis and *chi*-square test (Figure 9E-F). Further, the survival analysis showed that high expression levels of both MTSS1 and MTSS1-AS were most beneficial to overall survival of patients with PC, followed by the high expression level of either MTSS1 or MTSS1-AS; however, low expression levels of both MTSS1 and MTSS1-AS was unfavourable for the prognosis of patients with PC (Figure 9G). Moreover, the combination MTSS1 and MTSS1-AS showed an additive predictive value for overall survival compared with any individual target in the ROC curve analysis (Figure 9H). The immunohistochemical analysis confirmed that the protein levels of Myc and MZF1 inversely correlated with the expression levels of MTSS1-AS and MTSS1 (Figure 9I). Together, these results further indicated that the Myc/MTSS1-AS/MZF1/MTSS1 signalling contributed to the metastasis and progression of PC (Figure 9J).

## Discussion

Although numerous studies revealed that acidic extracellular microenvironments induced the migration and invasion of cancer cells, the underlying mechanism was not fully understood 41. Recently, the diverse functions of lncRNAs in cancer development have become a research hotspot. However, an association of lncRNAs with acidic microenvironments-induced metastasis in cancers has not yet been established. In our present study, the expression level of MTSS1-AS significantly reduced in acidic PC cells, and the overexpression of MTSS1-AS remarkably inhibited the acidity-induced metastasis of PC cells. Moreover, MTSS1-AS was downregulated in PC tissues and correlated with metastasis and poor prognosis. Therefore, our study was novel in identifying a pivotal lncRNA involved in the acidity-induced metastasis of PC.

Recent studies indicated that antisense lncRNA regulated the expression of the sense gene through various mechanisms, such as providing a physical barrier against posttranscriptional interactions, triggering RNA interference, and so forth 42, 43. For example, forkhead box protein C2 antisense RNA 1 (FOXC2-AS1) regulated the expression of FOXC2 by increasing FOXC2 mRNA stability via the forming of an RNA double-stranded structure in the overlapping region 44. Similarly, keratin 7 (KRT7) antisense RNA 1 (KRT7-AS1) promoted the stability of KRT7 mRNA by forming an RNA-RNA hybrid, which enhanced the expression of KRT7 45. Meanwhile, SATB homeobox 2 antisense RNA 1 (SATB2-AS1) directly bound to WD repeat domain 5 and growth arrest and DNA damage inducible 45 alpha, *cis*-activating SATB2 transcription via mediating histone H3 lysine 4 tri-methylation deposition, and DNA demethylation of the promoter region of SATB2 46. The present study demonstrated that MTSS1-AS relieved the inhibitory effect of MZF1 on MTSS1 transcription by inducing the STUB1-dependent degradation of MZF1 protein. Therefore, the results provided specific mechanisms for antisense RNA-regulating sense gene expression. Meanwhile, the overexpression of MTSS1 significantly inhibited the acidity-induced metastasis of PC cells, and the depletion of MTSS1 reversed the inhibitory effect of MTSS1-AS on PC cell metastasis. Consistent with a previous study 26, the present study also showed that MTSS1 was significantly downregulated in PC tissues compared with peritumoral tissues. Moreover, the expression level of MTSS1 positively correlated with the expression of MTSS1-AS and associated with poor overall survival. Therefore, the results of this study indicated that MTSS1 was a critical target of MTSS1-AS and functioned as an inhibitor in PC metastasis.

Since MTSS1-AS is mainly located in the nuclei of PC cells, it was hypothesized that MTSS1-AS might regulate MTSS1 transcription by influencing the binding between the transcription factor and promoter area of MTSS1 gene. Online software catRAPID and JASPAR were used to predict the protein potentially interacting with MTSS1-AS and MTSS1 promoter. The interaction analysis of catRAPID and JASPAR suggested a superior combination potential of MZF1 on MTSS1-AS and MTSS1 promoter. MZF1 has been shown to serve as a transcription factor targeting excision repair cross-complementation group 1, which was involved in the development of drug resistance of human ovarian cancer 47. MZF1 induced the migration and invasion of colorectal and cervical cancer cells by transcriptionally enhancing the expression of AXL receptor tyrosine kinase 48. Other studies also showed that the stabilization of MZF1 enhanced EMT by upregulating the expression of N-cadherin 49. Nevertheless, MZF1 inhibited cell migration and metastasis of human cervical cancer by repressing matrix metallopeptidase 2 transcription 50. Therefore, it was speculated that MZF1 might mediate the regulation of MTSS1-AS on MTSS1 transcription. In the present study, the interaction of MZF1 with MTSS1-AS was validated by RNA pull-down and RIP assay. Furthermore, the ChIP assay and dual luciferase reporter assay verified that MZF1 functioned as an inhibitory factor to repress MTSS1 transcription. Nevertheless, the results of this study showed that the binding between MZF1 and MTSS1 promoter was enhanced under acidity conditions, but obviously decreased after the overexpression of MTSS1-AS. Accordantly, the expression of MTSS1 was inhibited by the overexpression of MZF1, but increased after MZF1 depletion. Meanwhile, the overexpression of MZF1 abolished the inhibitory effect of the MTSS1-AS/MTSS1 pathway on the acidity-induced metastasis of PC cells. Therefore, the findings of this study implied that the interaction of MTSS1-AS and MZF1 impeded the binding of MZF1 with the MTSS1 promoter, which consequently relieved the inhibitory effect of MZF1 on MTSS1 transcription. Thereby, the downregulation of MTSS1-AS led to the enhanced inhibition of MZF1 on the MTSS1 transcription of PC cells under acidic conditions.

Then, the potential mechanism by which MTSS1-AS regulated the interaction between MZF1 and MTSS1 promoter was thoroughly investigated. The results showed that the overexpression and depletion of MTSS1-AS remarkably increased or decreased the expression of MZF1 at the protein level but not at the mRNA level, indicating that MTSS1-AS regulated the expression of MZF1 at the posttranscriptional level. Numerous studies revealed that lncRNAs interacted with protein and regulated its stability. For instance, hypoxia-induced lncRNA-AC020978 directly interacted with pyruvate kinase M2 (PKM2) and enhanced the protein stability of PKM2, promoting the proliferation and glycolysis of non-small cell lung cancer cells 51. A previous study also showed that lncRNA-CF129 induced ubiquitination-dependent p53 degradation by enhancing the interaction between p53 and makorin ring finger protein 1 in PC 52. Therefore, whether MTSS1-AS regulated the expression of MZF1 by modulating its stability was investigated. Coincidently, this study revealed that MZF1 stability was obviously enhanced in PC cells under acidic conditions, but remarkably impaired by the overexpression of MTSS1-AS. Moreover, it verified that MTSS1-AS induced the ubiquitination-dependent degradation of MZF1 protein. Previous studies showed that MZF1 protein stability was regulated by E3 ubiquitin-protein STUB1 via the ubiquitin-proteasome pathway 35. Therefore, it was speculated that STUB1 was involved in the mechanism by which MTSS1-AS modulated MZF1 stabilization. In accordance with this, the present study demonstrated that the interaction between STUB1 and MZF1 reduced in PC cells under acidic conditions but enhanced by the overexpression of MTSS1-AS. In addition, the depletion of STUB1 rescued MZF1 in PC cells, which was inhibited by the overexpression of MTSS1-AS. On the contrary, the overexpression of STUB1 decreased the expression of MZF1 in acidic PC cells. Taken together, the results of this study suggested that MTSS1-AS destabilized MZF1 via STUB1-dependent ubiquitination, abolishing the inhibitory effect of MZF1 on MTSS1 transcription. Meanwhile, the mechanism by which antisense lncRNA upregulated the sense gene expression by promoting the degradation of transcription factors was different from that reported in previous studies, which provided novel evidence for a “close relationship” between antisense lncRNA and their counterpart mRNA.

Bio-information analysis was used to identify the putative transcriptional factor that regulated the expression of MTSS1-AS to further clarify the mechanism by which extracellular acidity affected the expression of MTSS1-AS. Numerous studies found that Myc activated transcription through E-box Myc binding sites but represses transcription through a mechanism dependent on initiator (Inr) elements 38, 53. In addition, Myc is known as a key protein in coupling diverse cellular programs of cancer cells with their microenvironment. In addition, previous studies indicated a significant Myc upregulation of cancer cells under acidic conditions 37. Similarly, this study also showed an obvious increase in the expression of Myc in PC cells under acidic conditions. In addition, the bio-information analysis suggested an Inr element located in the promoter area of MTSS1-AS. Coincidently, the binding between Myc and Inr elements of MTSS1-AS was validated by the ChIP assay and the inhibitory effect of Myc on MTSS1-AS promoter activity was confirmed by dual-luciferase reporter assay. Meanwhile, the results showed that the binding between Myc and MTSS1-AS promoter was significantly enhanced under acidic condition, leading to obvious transcriptional repression of MTSS1-AS. On the contrary, Myc knockdown remarkably increased the transcription of MTSS1-AS and inhibited the acidic-induced metastasis of PC cells. Therefore, the present study suggested that Myc was the key transcription factor responsible for the modulation of the MTSS1-AS/MTSS1 pathway and acidity-induced metastasis in PC cells.

The formation of the feedback loop was essential in cancer progression 19, 54. Meanwhile, the involvement of Myc has been indicated in certain positive or negative feedback loops. For example, the Myc/GCN2/eIF2α negative feedback loop was shown to limit protein synthesis and prevent Myc-dependent apoptosis in colorectal cancer, while the FGFR3/Myc positive feedback loop provided new opportunities for targeted therapies in bladder cancer 55, 56. Also, previous studies reported that a reciprocal feedback loop between the antisense lncRNA of glutaminase and Myc was involved in the proliferation and invasion of PC during glutamine deprivation 57. Tsai et al. demonstrated that the expression of Myc was transcriptionally activated by dose-dependent MZF1 expression in lung adenocarcinoma 40. As MZF1 was regulated by MTSS1-AS, whether MTSS1-AS could regulate the expression of Myc, which formed a reciprocal feedback loop, was investigated. Coincidently, the results showed that the downregulation or overexpression of MZF1 dramatically decreased or increased the expression of Myc in PC cells. Afterward, the expression of Myc was inhibited or promoted after the overexpression or knockdown of MTSS1-AS under both normal and acidic conditions. The ChIP and dual luciferase reporter assays showed that the acidic-enhanced interaction between MZF1 and Myc promoter was inhibited after the overexpression of MTSS1-AS but was rescued by MZF1 re-expression. Meanwhile, the depletion of MTSS1-AS increased the interaction between MZF1 and Myc promoter but was obviously relieved by the knockdown of MZF1. The Transwell assay further revealed that the Myc-induced invasion of PC cells was significantly inhibited by the overexpression of MTSS1-AS or downregulation of MZF1. Conversely, the acidic-induced invasion of PC cells was inhibited by the downregulation of Myc, but restored by the downregulation of MTSS1-AS or overexpression of MZF1. Therefore, the findings of this study provided evidence that MTSS1-AS reciprocally regulated the expression of Myc via modulating MZF1-dependent transcription in PC cells.

## Conclusions

Together, this study implicated an extracellular acidity-triggered a reciprocal feedback loop between Myc and MTSS1-AS, which was involved in the metastasis of PC by regulating the expression of MTSS1. It suggested that MTSS1-AS was a potentially valuable biomarker and therapeutic target for PC.

## Supplementary Material

Supplementary figures and tables.

## Abbreviations

Act-D: Actinomycin D
AUC: area under curve
ChIP: Chromatin immunoprecipitation
CHX: cycloheximide
Co-IP: Co-immunoprecipitation
EMT: epithelial-mesenchymal transition
FISH: Fluorescence *in situ* hybridization
FOXC2-AS1: forkhead box protein C2 antisense RNA 1
FPKM: fragments per kilobase million
HPDE: human pancreatic duct epithelial
IHC: Immunohistochemistry
IκBα: NFKB inhibitor alpha
Inr: initiator
KRT7: Keratin 7
KRT7-AS: KRT7 antisense RNA 1
LncRNA: long non-coding RNA
MTSS1: metastasis suppressor 1
MTSS1-AS: antisense lncRNA of metastasis suppressor 1
Myc: MYC proto-oncogene
MZF1: myeloid zinc finger 1
NCCN: National Comprehensive Cancer Network
NP: normal peritumoral
PC: pancreatic cancer
PKM2: pyruvate kinase M2
RIP: RNA immunoprecipitation
RNA-seq: RNA sequencing
ROC: receiver operating characteristic
RSEM: RNA-seq by Expectation Maximization
SATB2-AS1: SATB homeobox 2 antisense RNA 1
SD: standard deviation
STUB1: STIP1 homology and U-box containing protein 1
