# Supplementary material for: A reciprocal feedback of Myc and lncRNA MTSS1-AS contributes to extracellular acidity-promoted metastasis of pancreatic cancer
Source: Theranostics. 2020 Aug 8;10(22):10120–40. doi: 10.7150/thno.49147 (PMC7481418; doi:10.7150/thno.49147)
Supplement: Supplementary file 1 — Supplementary figures and tables. [file thnov10p10120s1.pdf]

A reciprocal feedback of Myc and lncRNA MTSS1-AS contributes to extracellular acidity–promoted metastasis of pancreatic cancer

Supplementary Methods

Transfection

MTSS1-AS siRNA, MTSS1 siRNA, MZF1 siRNA, Myc siRNA, STUB1 siRNA and negative controls siRNA were provided by RiboBio (Guangzhou, China) and transfected with a final concentration of 50 nM. MTSS1-AS, MZF1, MTSS1, Myc overexpression and empty vector plasmid were purchased from GeneChem (Shanghai, China) and transfected with 1.6μg per 12 well plates. For stable transfection, MTSS1-AS and Myc overexpression plasmid were constructed into lentiviral vector and empty lentiviral vectors were as negative control. Lipofectamine 2000 (Invitrogen, Carlsbad, CA, USA) and Opti-MEM (Gibco) was used for cell transfection refer to the manufacturer's instructions. The transfection efficiency was identified via observing fluorescence intensity. Cells were cultured at normal medium or acidic medium for additional 48 h after transfection until collected for further assay. The sequences of siRNAs are shown in Table S3.

qRT-PCR

Total RNA from either cell lines or tissue samples was isolated using Trizol RNAiso Plus (Takara, Dalian, China). Reverse transcriptions of all lncRNA and mRNAs were performed by PrimeScript™ RT reagent Kit (Takara) based on provided directions. Reaction procedures of reverse transcriptions were as follows: 37 °C for 15 min; 85 °C for 5 s; 4 °C for 10 min. PCR based on provided directions of SYBR®Premix Ex Taq™ (TaKaRa) and was conducted using StepOne-Plus System (Applied Biosystems, USA). β-actin was served as loading control. Each sample was done in triplicate. $2^{-\Delta\Delta Ct}$ was calculated to present the relative expression level of gene. Primer sequences are given in Table S1.

Cell proliferation assay

Cells were seeded into 96-well plates at a density of 2000 cells per well and incubated at 37 °C for 1 to 5 days. Then, cells were incubated with MTT solution (0.5 mg/mL) at 37 °C for 4 h and the media was replaced by 150 μL DMSO. A microplate reader was used to detect the absorbance at 570 nm. All experiments repeated three times independently and there were five samples per group.

Migration and invasion assay

The migratory ability of PANC-1 and BxPC-3 cells was assessed by wound healing assay. Stable transfected cells were seeded in 12-well plates to form the single confluent cell layer. Then cells were cultured in FBS-free (without Fetal Bovine Serum) medium. We observed the healing of the wound at 0 h, 12 h and 24 h after would scratching with microscope. Transwell migration chambers with 8-μm pore polycarbonate membranes (Corning, Shanghai, China) were pre-coated with a thin layer of Matrigel Basement Membrane Matrix (BD Biosciences, Shanghai, China). Suspensions of 5×10^4 cells in 100 μL medium were added to the upper chamber. Medium with 30% fetal bovine serum in the lower chamber was used as chemoattractant. After incubating 48 h, cells on the membrane of lower chamber were fixed with 4% paraformaldehyde, stained with 0.1% crystal violet, and counted under a microscope in nine random fields. Each experiment was performed in triplicate.

Western blot analysis

Treated cells were lysed with lysis buffer combined with protease inhibitors to extract total protein. The prepared protein samples were electrophoretic in SDS-PAGE gel, and were then transferred to PVDF membranes (Millipore, USA). After blocking, respective primary antibodies were incubated with protein bands at proper temperature and time, and secondary antibodies were incubated subsequently. Protein bands were washed ultimately, and band signals were visualized using ECL reagent (Servicebio, China) as well as collected by ChemiDocTm XRS Molecular Imager System (Bio-Rad). Antibodies for the Western blot were as follows: MTSS1 (Santa Cruz Biotechnology, Shanghai, China), MZF1 (Santa Cruz Biotechnology, Shanghai, China), Myc (Cell Signaling, Danvers, MA, USA), STUB1 (Santa

Cruz Biotechnology, Shanghai, China), Ubiquitin (Proteintech, WuHan, China), β-actin (Proteintech, WuHan, China), GAPDH (Proteintech, WuHan, China). Secondary antibodies were HRP-labeled goat anti-mouse/rabbit IgG(H+L) purchased from CST.

Luciferase reporter assay

MTSS1, MTSS1-AS, Myc promoter region (2.0 kb sequence upstream of transcription initiation site) containing putative binding area (wild type, WT) or mutant area (mutant type, MUT) was constructed into pGL3-based vectors (nominated as pGL3-MTSS1, pGL3-MTSS1-AS or pGL3-Myc), and transfected in PC cells. For MTSS1, the transfected cells were co-transfected with pcDNA-MZF1 (0.2 μg) or pcDNA-MTSS1-AS (0.2 μg) and corresponding negative control respectively in 96-well plate, and then cultured under normal or acidic conditions. For MTSS1-AS, the transfected cells were co-transfected with Myc siRNA (50 nM) or negative controls siRNA and then cultured under normal or acidic conditions. For Myc, the transfected cells were co-transfected with pcDNA-MZF1 (0.2 μg), pcDNA-MTSS1-AS (0.2 μg), MZF1 siRNA (50 nM), MTSS1-AS siRNA and corresponding negative control respectively in 96-well plate, and then cultured under normal or acidic conditions. The luciferase activities were measured using dual luciferase reporter system (Promega, Madison, WI, USA). Briefly, the prepared samples were incubated with firefly luciferase detection reagent or renilla luciferase detection reagent for 10 min at room temperature. Fluorescence signal and intensity were measured using fluorescence microplate reader. Firefly luciferase activities were normalized by renilla luciferase activities. All experiments repeated three times independently and there were five samples per group.

Table S1. The sequence of PCR primers.

Primer	Sequence
MTSS1-AS	Forward: 5'-AATGCTCAGGGAACACCTGG -3'
	Reverse: 5'-AGCACAACACTCTGAGAGCC-3'
MZF1	Forward: 5'-CGATGTATGTGGCAAGGT-3'
	Reverse: 5'-CACTCTCCGATGCTCTTC-3'
MTSS1	Forward: 5'-GGAATCTGACCACTATACTGT-3'
	Reverse: 5'-CTGTTATGACCTGACTGCTA-3'
Myc	Forward: 5'-GGCTCCTGGCAAAAGGTCA -3'
	Reverse: 5'-CTGCGTAGTTGTGCTGATGT -3'
β-actin	Forward: 5'-ATGTGGCCGAGGACTTTGAT -3'
	Reverse: 5'-AGTGGGGTGGCTTTTAGGATG-3'
ChIP primers for MTSS1	
Site 1	Forward: 5'-GACAGCACACCAATGTTACAGAAGT-3'
	Reverse: 5'-CACAACCTACTAACTGTATGCCTTT-3'
Site 2	Forward: 5'-CAATCTGGCTCTACAAGCAGTCATC-3'
	Reverse: 5'-GGTCTGGCTTTGAAAACTTTATGGA-3'
Site 3	Forward: 5'-TTGATAGTTAGAATACCAATAGTAT-3'
	Reverse: 5'-GGTCTGGCTTTGAAAACTTTATGGA-3'
ChIP primers for MTSS1-AS	Forward: 5'-AAGAGCCATGTGTTGGTGCA-3'
	Reverse: 5'-TGGGGGTAGGGTAGTGATAA-3'
ChIP primers for Myc	Forward: 5'-CGGGGCAGGAGGGGCGGTAT-3'
	Reverse: 5'-GCATTTGTTGGGGGGAGTCA-3'

Table S2. The sequence of siRNA.

SiRNA Targets	Sequences
MTSS1-AS-siRNA#1 sense	5'-ACACAACCAAAUCUGAUUU-3'
MTSS1-AS-siRNA#2 sense	5'-CUCCCAGGACAGAGGGACA-3'
MTSS1-AS-siRNA#3 sense	5'-AUGAAACAUUUCACAAACUC-3'
MTSS1-siRNA#1 sense	5'-GCUUCUGAUUACAGCUGGU-3'
MTSS1-siRNA#2 sense	5'-CGACACACAGCUCUCUAAA-3'
MTSS1-siRNA#3 sense	5'-UCUCUGGCAACUGAGAGAA-3'
MZF1-siRNA#1 sense	5'-CUGGCCUCAGAUACCUGAC-3'
MZF1-siRNA#2 sense	5'-GUGCAGGGCCAGGAGGUCC-3'
MZF1-siRNA#3 sense	5'-GCCCAGCUCCGAGAGCUGU-3'
Myc-siRNA#1 sense	5'-AACAGAAAUGUCCUGAGCAA-3'
Myc-siRNA#2 sense	5'-GGACUAUCCUGCUGCCAAGAG-3'
Myc-siRNA#3 sense	5'-GUCCUGAGCAAUCACCUAUGA-3'
NC-siRNA sense	5'-UUCUCCGAACGUGUCACGUUU-3'

FISH probe: 5'-ACCAAGAGTGTCCACTGGCGGGGGGCACACGGT-3'

Table S3. Predicted proteins which could interact with MTSS1-AS via catRAPID.

Protein	Z-score	Discriminative Power (%)	Domain	Protein	Z-score	Discriminative Power (%)	Domain
DDX1	1.52	99	yes	RFX2	0.6	92	yes
CACO1	1.45	98	yes	RXRB	0.6	92	yes
GZF1	1.42	98	yes	CEBPB	0.6	92	yes
KIF2C	1.4	98	yes	NOL4	0.61	92	yes
PO6F2	1.26	98	yes	PINX1	0.57	92	yes
ZN408	1.19	98	yes	PTF1A	0.58	92	yes
CTCF	1.15	98	yes	RED1	0.58	92	yes
RABX5	1.1	97	yes	Z280A	0.58	92	yes
SSRP1	1.09	97	yes	LMX1B	0.58	92	yes
PLAK	1.08	97	yes	ZBT7B	0.58	92	yes
DDX4	1.05	97	yes	PROX1	0.55	91	yes
GLU2B	1.07	97	yes	ABRA	0.56	91	yes
SCML2	0.98	97	yes	MTER2	0.57	91	yes
HDX	0.96	97	yes	MKRN3	0.55	91	yes
SHOX2	0.95	97	yes	**MZF1**	0.56	91	yes
FOXP2	0.95	97	yes	ELAV4	0.56	91	yes
EZH2	0.92	97	yes	NELFE	0.56	91	yes
PO4F3	0.93	97	yes	ATX1L	0.56	91	yes
CPSF3	0.91	96	yes	ANXA1	0.57	91	yes
TBX3	0.9	96	yes	SATB2	0.54	91	yes
TE2IP	0.86	96	yes	NDF6	0.55	91	yes
TS101	0.83	96	yes	FUBP2	0.52	91	yes
JUND	0.85	96	yes	DHX58	0.52	90	yes
DEK	0.83	96	yes	HNRPM	0.49	89	yes
FOXO3	0.82	96	yes	KHDR3	0.39	84	yes
TRM1L	0.79	96	yes	FUS	0.26	79	yes
MBNL1	0.71	95	yes	ESRP2	0.25	77	yes
PTCD	0.77	95	yes	ELAV1	0.25	77	yes
ZN366	0.78	95	yes	PTBP1	0.1	69	yes
RN5A	0.78	95	yes	ELAV2	-0.03	59	yes
HME2	0.76	95	yes	TRA2A	-0.04	56	yes
PEX14	0.78	95	yes	TIA1	-0.05	56	yes
DVL2	0.77	95	yes	RFOX2	-0.06	56	yes
HXD11	0.76	95	yes	FMR1	-0.07	54	yes
USF2	0.71	95	yes	TIAR	-0.09	52	yes

SKI	0.71	95	yes	HNRH3	-0.09	52	yes
PRP31	0.69	94	yes	HNRDL	-0.18	45	yes
FWCH1	0.69	94	yes	NOVA1	-0.26	37	yes
SRP54	0.7	94	yes	TRA2B	-0.33	32	yes
RFX3	0.69	94	yes	ROA1	-0.36	28	yes
PHAX	0.69	94	yes	TADBP	-0.37	28	yes
LARP4	0.67	94	yes	NOVA2	-0.39	26	yes
TBR1	0.68	94	yes	PCBP1	-0.4	26	yes
SUZ12	0.67	94	yes	DAZP1	-0.43	24	yes
TBX21	0.63	93	yes	HNRPF	-0.4	24	yes
RTF1	0.63	93	yes	HNRPD	-0.44	22	yes
NUCL	0.65	93	yes	SRSF9	-0.48	22	yes
ZBT48	0.63	93	yes	HNRH1	-0.47	22	yes
NKRF	0.64	93	yes	HNRPC	-0.51	20	yes
EDC3	0.63	92	yes	HNRH2	-0.54	17	yes
ARNT2	0.59	92	yes	PCBP2	-0.64	17	yes
TCF25	0.6	92	yes	SRSF1	-0.69	14	yes
FBXW7	0.61	92	yes	U2AF2	-0.76	14	yes
FOXJ1	0.59	92	yes	SFPQ	-0.79	14	yes
DDX17	0.61	92	yes	SRSF7	-0.82	14	yes
FOXP4	0.6	92	yes	LN28B	-0.84	14	yes
NUFP2	0.59	92	yes	SRSF2	-0.9	10	yes
KRR1	0.6	92	yes	SRS10	-0.9	10	yes
MTA1	0.62	92	yes	CELF1	-0.96	10	yes
HXA1	0.62	92	yes	A1CF	-1.12	10	yes

Table S4. Predicted binding data between the promoter region of MTSS1 and candidate transcription factor from JASPAR database.

Matrix ID	Name	Score	Relative score	Start	End	Strand	Predicted sequence
MA0056.2	MZF1	11.3223	0.901982136439	523	535	+	ATTATCCCCATTT
MA0057.1	MZF1(var.2)	10.9787	0.968367511124	849	858	+	GGATGGGGAA
MA0526.1	USF2	10.8744	0.921285497232	109	119	+	AGCATGTGGCA
MA0703.2	LMX1B	9.94169	0.884353057215	1522	1532	+	TCCTTAATTAT
MA0057.1	MZF1(var.2)	9.14525	0.910369427918	1736	1745	+	GTAGGGGTGG
MA0690.1	TBX21	9.12546	0.863329137916	1225	1234	+	GAGCTGTGAA
MA0703.1	LMX1B	9.08888	0.941099977644	1525	1532	+	TTAATTAT
MA0056.1	MZF1	9.08528	0.999999979352	768	773	+	TGGGGA
MA0056.1	MZF1	9.08528	0.999999979352	852	857	+	TGGGGA
MA0526.3	USF2	8.67359	0.830371642832	107	120	+	TAAGCATGTGGCAG
MA1566.1	TBX3	8.61926	0.905069228296	708	717	+	GATGTGTCAA
MA0056.1	MZF1	8.50962	0.973734687631	840	845	+	CGGGGA
MA0056.1	MZF1	8.50962	0.973734687631	1691	1696	+	CGGGGA
MA0056.1	MZF1	8.25214	0.961986739759	1799	1804	+	GGGGGA
MA0056.2	MZF1	8.11514	0.834563798493	291	303	+	AAAATGCCCAGAA
MA0157.1	FOXO3	8.09164	0.863939523932	65	72	+	GGCAAACA
MA0056.1	MZF1	7.93851	0.947676605646	894	899	+	AGGGGA
MA0600.1	RFX2	7.82651	0.803456423172	1430	1448	+	ATTCATTTAGCAACAGCAA
MA0690.1	TBX21	7.77219	0.841020789392	708	717	+	GATGTGTCAA
MA0466.2	CEBPB	7.6018	0.887775561605	1434	1443	+	ATTTAGCAAC
MA0056.2	MZF1	7.59226	0.823572102879	1036	1048	+	CCACTCCCCCCAG
MA0157.2	FOXO3	7.4599	0.857472116718	66	73	+	GCAAACAT
MA0157.2	FOXO3	7.4599	0.857472116718	274	281	+	GTAGACAT
MA0690.1	TBX21	7.41729	0.835170504424	601	610	+	AGGTTGTGAA
MA0057.1	MZF1(var.2)	7.22386	0.849591054597	1828	1837	+	GGAGGGAGAG
MA0057.1	MZF1(var.2)	7.13075	0.846645765548	176	185	+	GGCTGGGTAA
MA0794.1	PROX1	7.11541	0.834057980887	148	159	+	TAAGGCCCCTTA
MA0057.1	MZF1(var.2)	7.07185	0.844782811588	542	551	+	GGATGAGGAA
MA1566.1	TBX3	7.00107	0.864616088653	1225	1234	+	GAGCTGTGAA
MA0057.1	MZF1(var.2)	6.99324	0.842296198158	783	792	+	GCAGGGGTGA
MA0157.1	FOXO3	6.95915	0.825896916456	1344	1351	+	TGAAAACC
MA0057.1	MZF1(var.2)	6.71571	0.833517105488	1089	1098	+	CAAGGGTTAA
MA1566.1	TBX3	6.5478	0.853284721732	1759	1768	+	AGGGTGCCGA
MA0056.1	MZF1	6.46905	0.880629809697	545	550	+	TGAGGA

MA1566.1	TBX3	6.30294	0.847163437645	1115	1124	+	TAGGTGCCTA
MA0703.2	LMX1B	6.28645	0.826941072529	566	576	+	AGGTTAAGTAA
MA0593.1	FOXP2	6.02655	0.824352164985	272	282	+	AAGTAGACATA
MA0703.1	LMX1B	6.01677	0.849151006825	686	693	+	ACAATAAT
MA0057.1	MZF1(var.2)	5.99324	0.810663704782	838	847	+	TTCGGGGAGA
MA0056.1	MZF1	5.8934	0.85436449622	1798	1803	+	CGGGGG
MA0703.1	LMX1B	5.82739	0.843483031998	260	267	+	ATAATTTC
MA1566.1	TBX3	5.76711	0.83376827856	1809	1818	+	AGGGTGCGCT
MA0466.2	CEBPB	5.73282	0.862470012326	568	577	+	GTTAAGTAAC
MA0056.1	MZF1	5.63592	0.842616570105	1802	1807	+	GGAGGA
MA0690.1	TBX21	5.55838	0.804526905082	784	793	+	CAGGGGTGAT
MA0703.1	LMX1B	5.36169	0.829544469968	101	108	+	GAAATTTA
MA0703.1	LMX1B	5.28107	0.827131487504	689	696	+	ATAATGAT
MA0703.1	LMX1B	5.27934	0.82707966646	430	437	+	ATGATTAC
MA0056.1	MZF1	5.26193	0.825552683	332	337	+	CTGGGA
MA1566.1	TBX3	5.21058	0.819855353561	132	141	+	CAAGTGTCCA
MA1566.1	TBX3	5.13211	0.817893648362	857	866	+	AAGGTGGGTT
MA0703.1	LMX1B	5.12532	0.822469962692	1372	1379	+	GCAATCAG
MA0703.1	LMX1B	5.12532	0.822469962692	1547	1554	+	GCAATCAG
MA0466.2	CEBPB	5.09672	0.853857389602	993	1002	+	CTGACACAAG
MA0703.1	LMX1B	5.09216	0.821477313544	1252	1259	+	CAAATCAA
MA0703.1	LMX1B	5.09216	0.821477313544	1446	1453	+	CAAATCAA
MA1566.1	TBX3	5.05907	0.81606788428	1586	1595	+	TGGGCGTCTT
MA0703.1	LMX1B	5.00867	0.818978357527	569	576	+	TTAAGTAA
MA0056.1	MZF1	5.00445	0.813804735128	787	792	+	GGGTGA
MA0056.1	MZF1	5.00445	0.813804735128	831	836	+	GGGTGA
MA0056.1	MZF1	4.98938	0.813116987652	83	88	+	TGGGGT
MA0056.1	MZF1	4.98938	0.813116987652	92	97	+	TGGGGC
MA0056.1	MZF1	4.98938	0.813116987652	196	201	+	TGGGGC
MA0056.1	MZF1	4.98938	0.813116987652	365	370	+	TGGGGC
MA1566.1	TBX3	4.90422	0.812196769013	1668	1677	+	GGCGCGCCAC
MA0703.1	LMX1B	4.87565	0.814997042786	947	954	+	TCCATAAA
MA0703.1	LMX1B	4.85518	0.814384481227	520	527	+	ATCATTAT
MA0056.1	MZF1	4.84584	0.806567645738	179	184	+	TGGGTA
MA0056.1	MZF1	4.84584	0.806567645738	879	884	+	TGGGCA
MA0056.1	MZF1	4.84584	0.806567645738	1262	1267	+	TGGGCA
MA0703.1	LMX1B	4.79791	0.812670305038	1129	1136	+	TTAATATC
MA1566.1	TBX3	4.74942	0.808326917316	1936	1945	+	CGCGTGCGGC

MA1566.1	TBX3	4.71797	0.80754063074	1666	1675	+	TAGGCGCGCC
MA0703.1	LMX1B	4.71119	0.810074828581	675	682	+	GTATTAAA
MA0466.1	CEBPB	4.66163	0.868803267386	71	81	+	CATTGCTAAAG
MA1566.1	TBX3	4.5603	0.80359894625	1739	1748	+	GGGGTGGGCG
MA0703.1	LMX1B	4.53249	0.804726443022	413	420	+	TTGATAAG
MA0703.1	LMX1B	4.41548	0.801224262425	976	983	+	CCCATTAC
MA0703.1	LMX1B	4.40908	0.801032577369	1432	1439	+	TCATTTAG
MA0466.2	CEBPB	4.32872	0.843458816004	1535	1544	+	ATTTCGCTAT
MA0526.1	USF2	4.1019	0.833296393268	1149	1159	+	CTCATTTGATT
MA0492.1	JUND(var.2)	3.946	0.838227856249	540	554	+	ATGGATGAGGAAACT
MA0466.2	CEBPB	3.66875	0.834522999204	72	81	+	ATTGCTAAAG
MA0526.1	USF2	3.35429	0.823583262209	323	333	+	GCAATGTGCCT
MA0492.1	JUND(var.2)	3.03468	0.828933401244	1568	1582	+	AGTCCTGACATCAGA
MA0492.1	JUND(var.2)	2.92802	0.82784551023	472	486	+	ATCGATCAGCTCATG
MA0466.1	CEBPB	2.75523	0.84882007225	1534	1544	+	TATTTCGCTAT
MA0466.2	CEBPB	2.69096	0.821283924854	544	553	+	ATGAGGAAAC
MA0466.2	CEBPB	2.67211	0.821028657122	437	446	+	CTTACGAGAT
MA0526.1	USF2	1.55575	0.800216291677	17	27	+	GCCTGGTGGTC
MA0466.1	CEBPB	1.50456	0.835710356317	1123	1133	+	TATGTCTTAAT
MA0466.2	CEBPB	1.38619	0.803617658527	1124	1133	+	ATGTCTTAAT
MA0492.1	JUND(var.2)	1.3464	0.811714671769	360	374	+	TGAAATGGGGCCAGG
MA0466.2	CEBPB	1.29169	0.802338161144	1011	1020	+	ATTTTGCACT
MA0491.1	JUND	1.17664	0.825188531172	741	751	+	GATGAGTCGGA
MA0466.1	CEBPB	0.139928	0.821406094091	1144	1154	+	AGTTGCTCATT
MA0491.1	JUND	-0.514845	0.806380641224	886	896	+	AGTGATCCAGG
MA0466.1	CEBPB	-0.627593	0.813360831361	1893	1903	+	GGGTGCCCCAC
MA0466.1	CEBPB	-0.824497	0.811296865264	1433	1443	+	CATTTAGCAAC

Note: The value in the “Start” and “End” column represents the number of bases starting at -2000 nt in the promoter region of MTSS1. Only predicted binding data for “+” strand was shown.

Table S5. Correlation between MTSS1-AS and clinical characteristics of patient with pancreatic cancer

Characteristics	Number of cases	MTSS1-AS expression		*P* value
		High	low	
Total cases	132			
Gender				
Male	61	30	31	0.1006
Female	71	45	26	
Age				
<60	55	19	36	0.0905
≥60	77	38	39	
Tumor size(cm)				
<2	59	23	36	0.0706
≥2	73	40	33	
Histological Grade				
High/Moderate	86	50	36	0.1078
Low	46	20	26	
Lymphatic invasion				
Positive	56	23	33	0.0270*
Negative	76	46	30	
Vascular invasion				
Positive	43	15	28	0.0298*
Negative	89	49	40	
Distant Metastasis				
Positive	60	25	35	0.0108*
Negative	72	46	26	

Note: Downregulation of MTSS1-AS was significantly associated with lymphatic invasion ($P = 0.0270$), vascular infiltration ($P = 0.0298$) and distant metastasis ($P = 0.0108$), but not with patients' gender ($P = 0.1006$), age ($P = 0.0905$), tumour size (P=0.0706) and histological grade (P = 0.1078). (*$P < 0.05$).

Supplementary Figure legends

A **Homo sapiens chromosome 8, complete sequence**
GenBank: RP11-532M24.1

AGGATGATCCTTTTTAAAGTTGGGGAACCACTAGCCCAACATCGCCATGAGGGGAAATAGAGGCCGTGTCAGCC
CCAACCATCAAAGGAGACCTTTGTGCACTGGGTTTGCTGTAAATGACAGAAGCCACTTCAAAAGCCTCTTTGGAG
CTGTTCAGTAAAATGCTCAGGGAACACCTGGGCACACTCAGGCAAGATAAACCACACAGACACCTCGCAGACAG
ACCTCCCAGGACAGAGGGACAGCAACAAGACTCCAAAAGCAACTTGAAGTTTTGGCAGCATGATCATCCACAGA
GTGAAGTTCAACATTCAGTCAAGAAGAATCATTCTGATTACTTAATTGGAAGATCTGATACTTCTGAAATGAAACAT
TTCACAAACTCTACTGAAATAAGAAATACTTTTGATTTATTTTTTAAAAAATGAATCATTGGTTATGGAAAGAACTAT
ATGGTCTTGCAGACAGGACAAAGTTAAAGATGTTTTGCAAGCTTAACACAACCAAATCTGATTTGAGAATTATTTTG
AGGCTTGCAGCTCTGTTTGTGGCATAAATATATACATACATACATACGCAGGTATATATGTATGCAAAAATGCTTAT
TGACTGTGGAAATGACAGTTTAAGTCACTCTGTATTTCTTTTTTCCATGAGTCTAATATTGAATTACGATGATTCAA
AGGCTCTCAGAGTGTTGTGCTAACATCGTAAATGGCTAAACATCCAAAAAAGAAAACAATTAAAACACAACGCACA
CACAATTTTCTTAAAGTTGTGGAAAATTAGGTAGGCACAGTTGATTGTTTTGAGCTGGTAGAAAAACCTGGGCAGC
TAGCAGAGGTCTGGACAACCACGGAAAGCCCACAGCAAGGACCACACCGTGAGCAGCCACCAGAGATTTGGGA
TGGTAGACTTACCAAGAGTGTCCACTGGCGGGGGGGCACACGGTGAGAAAGTGCAGGATACACAATTGACACAA
AAAGGAAAATAATAAAGTCAAACTGGTATTCAAGCA

B **MTSS1-AS secondary structure**

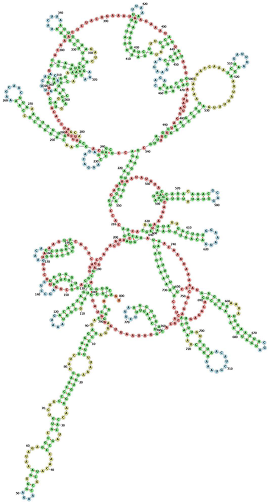

C Protein coding potential

Metric	Raw result	Interpretation
PRIDE reprocessing 2.0	0	non-coding
Lee translation initiation sites	0	non-coding
PhyloCSF score	-69.9486	non-coding
CPAT coding probability	5.96%	non-coding
Bazzini small ORFs	0	non-coding

In stringent set: yes

D

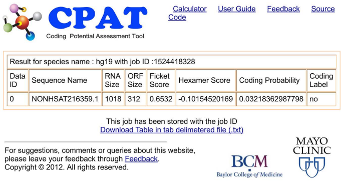

CPAT
Coding Potential Assessment Tool
Calculator User Guide Feedback Source Code

Result for species name : hg19 with job ID :1524418328							
Data ID	Sequence Name	RNA Size	ORF Size	Ficket Score	Hexamer Score	Coding Probability	Coding Label
0	NONHSAT216359.1	1018	312	0.6532	-0.10154520169	0.03218362987798	no

This job has been stored with the job ID
Download Table in tab delimetered file (.txt)

For suggestions, comments or queries about this website, please leave your feedback through Feedback.
Copyright © 2012. All rights reserved.

BCM Baylor College of Medicine
MAYO CLINIC

E

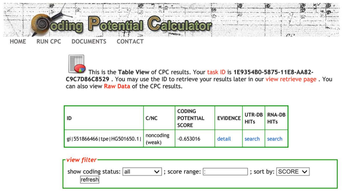

Coding Potential Calculator
HOME RUN CPC DOCUMENTS CONTACT

This is the **Table View** of CPC results. Your task ID is **1E9354B0-5875-11E8-AA82-C9C7D86C8529** . You may use the ID to retrieve your results later in our view retrieve page . You can also view Raw Data of the CPC results.

ID	C/NC	CODING POTENTIAL SCORE	EVIDENCE	UTR-DB HITs	RNA-DB HITs
gi|551866466|tpe|HG501650.1|	noncoding (weak)	-0.653016	detail	search	search

view filter
show coding status: all ; score range: : ; sort by: SCORE
refresh

Figure S1. The characteristic of MTSS1-AS. (A) The whole sequence of MTSS1-AS was showed from NCBI (https://www.ncbi.nlm.nih.gov). **(B)** Prediction of MTSS1-AS secondary structure provided by NONCODE (http://www.noncode.org) based on minimum free energy (MFE) and partition function. Colour scale indicates the confidence for the prediction for each base with shades of red indicating strong confidence. **(C-E)** The potential coding capabilities of MTSS1-AS evaluated by NONCODE **(C)**, Coding Potential Assessment Tool (http://lilab.research.bcm.edu/cpat) **(D)** and Coding Potential Calculator (http://cpc.cbi.pku.edu.cn) **(E)**.

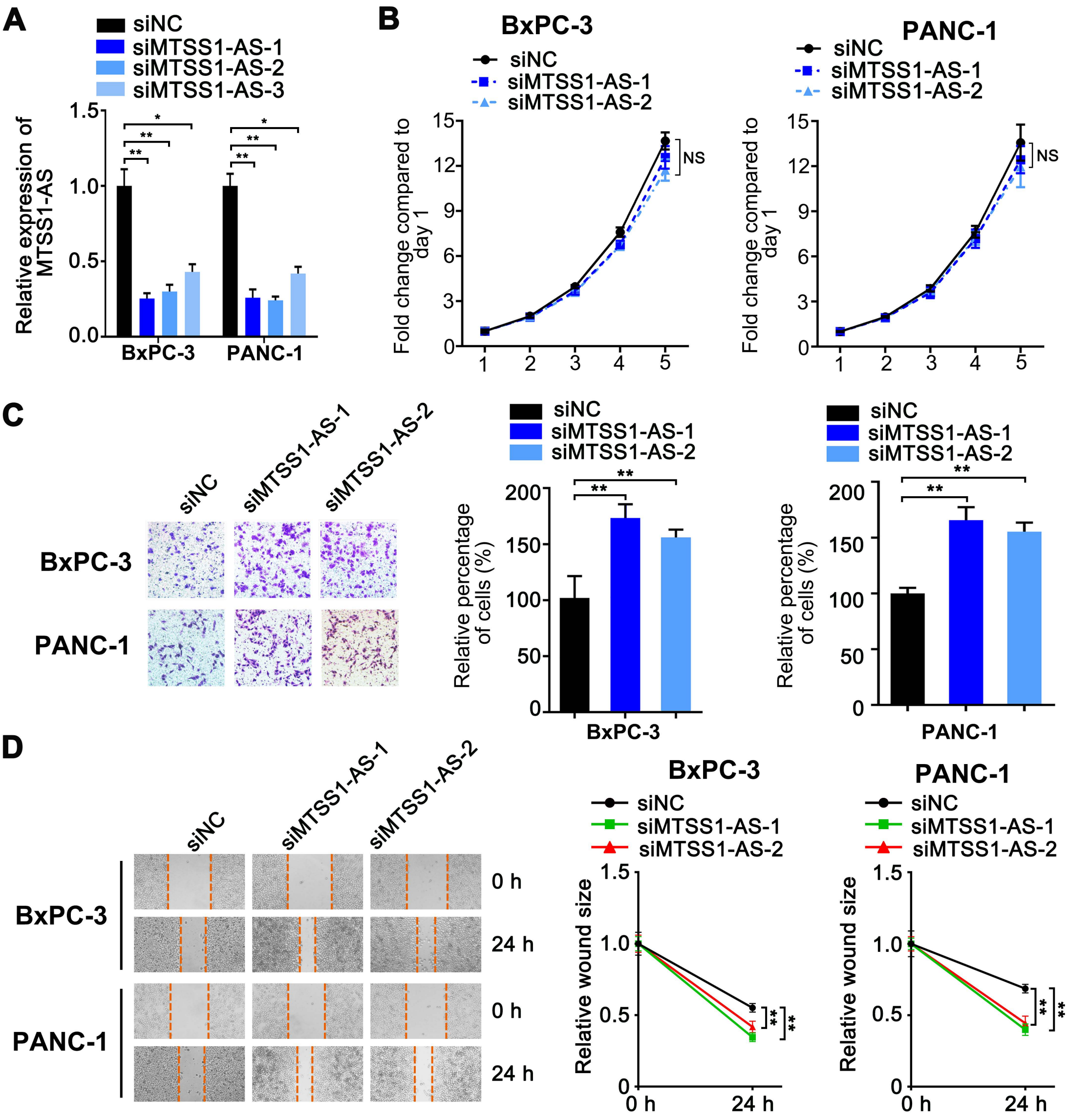

A
siNC
siMTSS1-AS-1
siMTSS1-AS-2
siMTSS1-AS-3
Relative expression of MTSS1-AS
BxPC-3
PANC-1
B
BxPC-3
PANC-1
Fold change compared to day 1
NS
C
BxPC-3
PANC-1
Relative percentage of cells (%)
D
0 h
24 h
Relative wound size

Figure S2. Inhibition of MTSS1-AS increased migration and invasion of PC cells. (A) The qRT-PCR analyzed the knockdown efficiency of MTSS1-AS by the three siRNAs. In following experiments, BxPC-3/PANC-1 cells were transfected with selected MTSS1-AS siRNAs (siMTSS1-AS-1, siMTSS1-AS-2) or the negative control siRNA. **(B)** Proliferation of MTSS1-AS knockdown BxPC-3/PANC-1 cells were measured by MTT assays for 5 days. **(C)** Invasion ability of MTSS1-AS knockdown BxPC-3/PANC-1 cells were assessed by Transwell assay. Representative images (Left) and relative percentage of invaded cells (Right) are shown. **(D)** Migration ability of BxPC-3/PANC-1 cells were assessed by wound healing assay. Representative images (Left) and relative wound size (Right) are shown. All data were presented as means ± SD of at least three independent experiments. Values are significant at $*P < 0.05$, $**P < 0.01$ as indicated. NS means the difference is not significant.

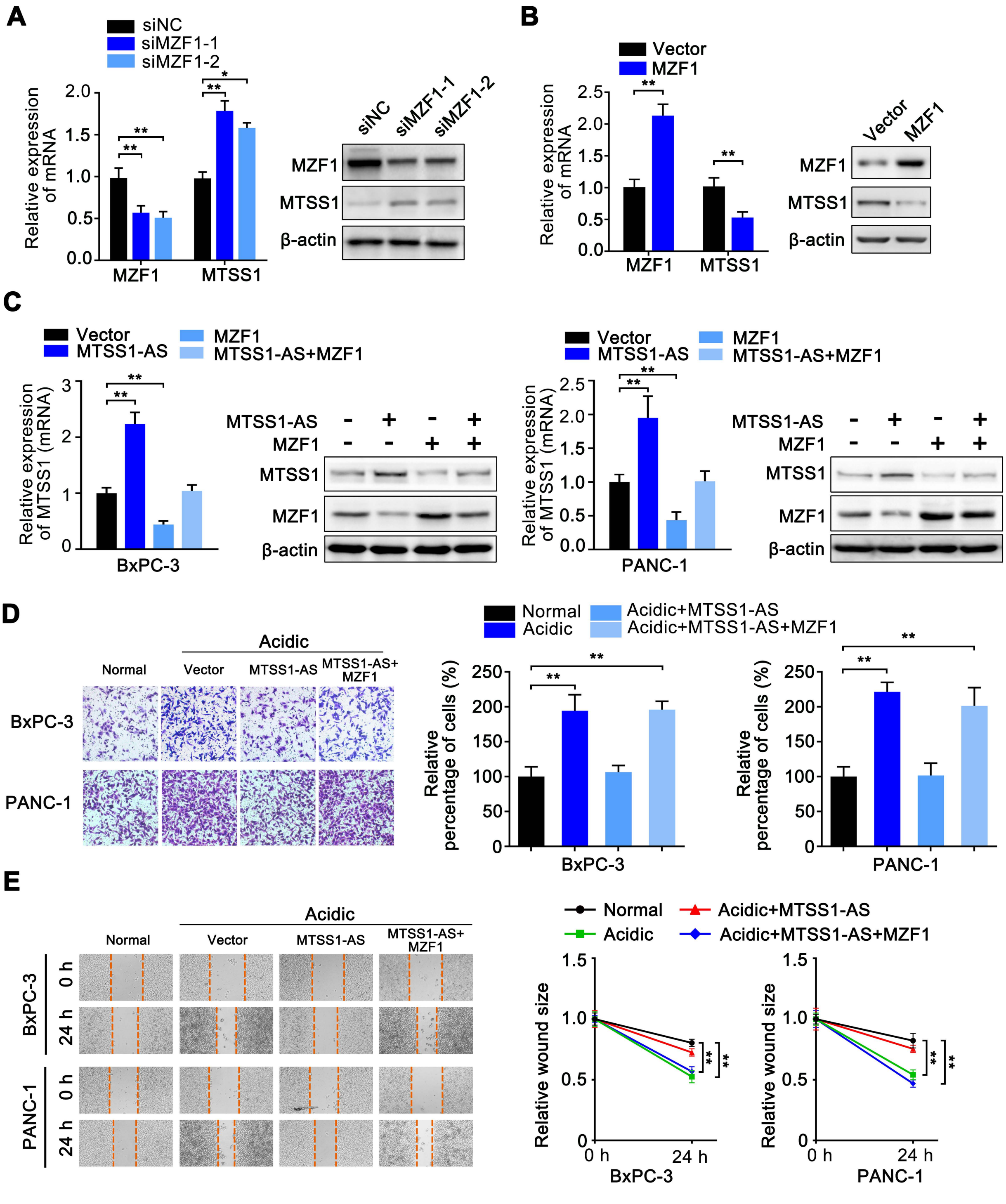

A
siNC
siMZF1-1
siMZF1-2
Relative expression of mRNA
MZF1
MTSS1
β-actin
B
Vector
MZF1
MTSS1
C
MTSS1-AS
MTSS1-AS+MZF1
Relative expression of MTSS1 (mRNA)
BxPC-3
PANC-1
D
Acidic
Normal
Acidic+MTSS1-AS
Acidic+MTSS1-AS+MZF1
Relative percentage of cells (%)
E
0 h
24 h
Relative wound size

Figure S3. MZF1 participated in the MTSS1-AS-mediated regulation of MTSS1. (A) Expression of MZF1 and MTSS1 was detected by qRT-PCR and Western blot analysis in BxPC-3 cells transfected with MZF1 siRNAs (siMZF1-1, siMZF1-2) or the negative control siRNA (siNC). **(B)** Expression of MZF1 and MTSS1 was detected by qRT-PCR and western blot analysis in BxPC-3 cells transfected with MZF1 overexpression plasmid (MZF1) or empty vector plasmid (Vector). **(C)** MTSS1 mRNA and protein levels were assessed by qRT-PCR and Western blot analysis in BxPC-3/PANC-1 cells transfected with vector, MTSS1-AS or/and MZF1 overexpression plasmid. **(D-E)** After co-transfection with pcDNA-MTSS1-AS plasmid or/and pcDNA-MZF1 plasmid as well as vector plasmid respectively, the invasion **(D)** and migration **(E)** ability of indicated transfected BxPC-3/PANC-1 cells were measured by Transwell and wound healing assays. **(D)** Representative images (Left) and relative percentage of invaded cells (Right) are shown. **(E)** Representative images (Left) and relative wound size (Right) are shown. All data were presented as means ± SD of at least three independent experiments. Values are significant at *$P < 0.05$, **$P < 0.01$ as indicated.

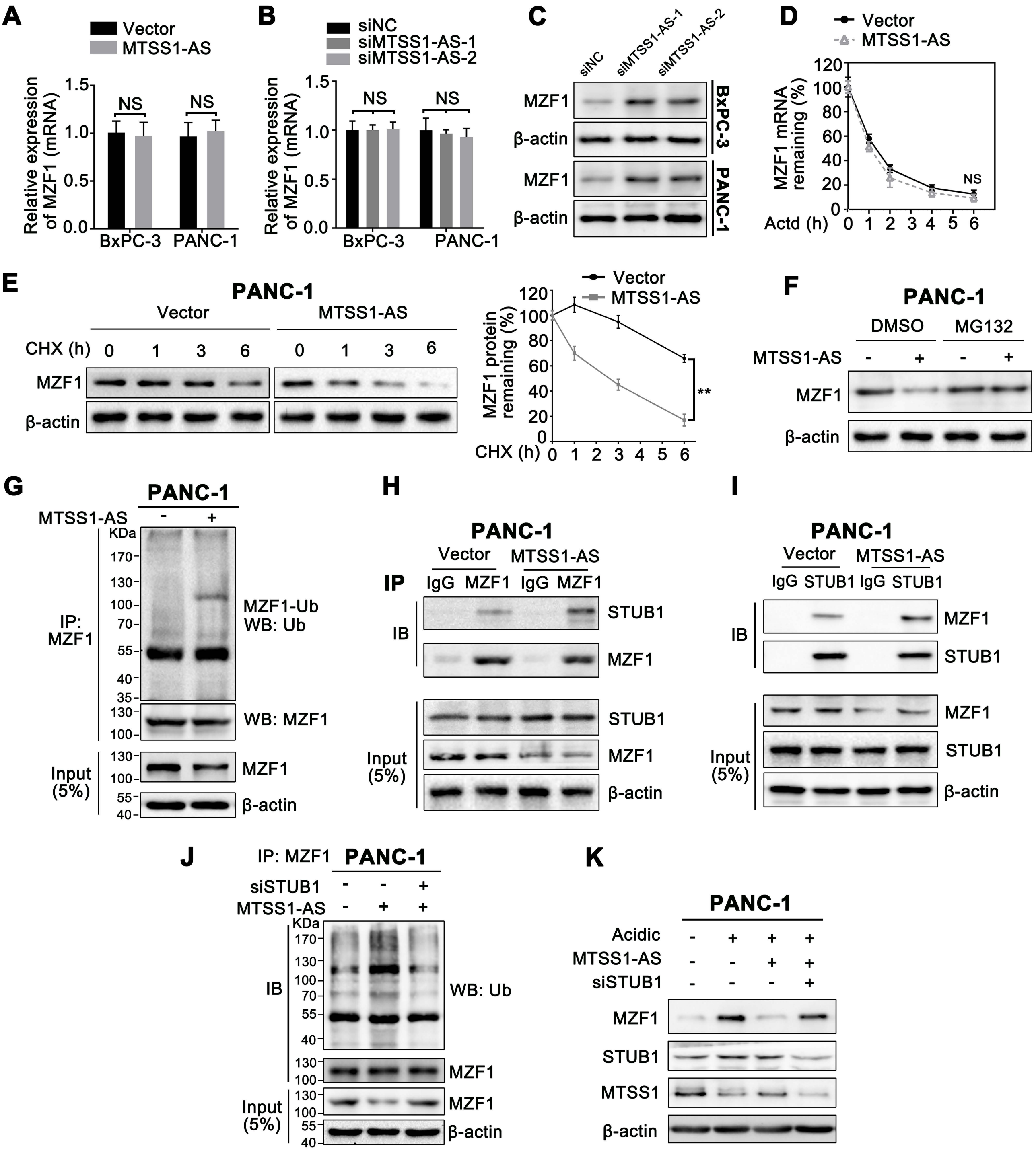

A
Vector
MTSS1-AS
Relative expression of MZF1 (mRNA)
NS
BxPC-3
PANC-1
B
siNC
siMTSS1-AS-1
siMTSS1-AS-2
C
MZF1
β-actin
BxPC-3
PANC-1
D
MZF1 mRNA remaining (%)
Actd (h)
E
PANC-1
CHX (h)
MZF1 protein remaining (%)
**
F
DMSO
MG132
G
IP: MZF1
MZF1-Ub WB: Ub
WB: MZF1
Input (5%)
KDa
H
IP
IgG
IB
STUB1
I
J
IP: MZF1
siSTUB1
WB: Ub
K
Acidic
MTSS1

Figure S4. MTSS1-AS inhibited MZF1 protein level by promoting ubiquitination-dependent degradation of MZF1 by STUB1. (A) MZF1 mRNA levels were assessed by qRT-PCR in MTSS1-AS overexpression BxPC-3/PANC-1 cells. **(B-C)** MZF1 mRNA **(B)** and protein **(C)** levels were assessed by qRT-PCR and Western blot analysis in MTSS1-AS knockdown BxPC-3/PANC-1 cells. **(D)** Total cellular RNA, harvested at 0, 1, 2, 3, 4, 5, and 6 h after the addition of Act-D in MTSS1-AS overexpression BxPC-3 cells, was quantified by qRT-PCR respectively. **(E)** PANC-1 cells transfected using Vector or MTSS1-AS overexpression plasmid for 48 h, were exposed to 100 μg/mL cycloheximide (CHX) for the indicated periods of time. MZF1 protein levels were measured (Left). The line chart indicates relative quantification (Right). **(F)** PANC-1 cells transfected with MTSS1-AS overexpression plasmid or vector were treated with MG132 (20 nM) or DMSO for 3 h before Western blot analysis. **(G)** Whole cell lysates of PANC-1 cells transfected with the MTSS1-AS overexpression plasmid or vector were immunoprecipitated with MZF1 antibody. MZF1 ubiquitination using anti-ubiquitin antibody was analyzed in the cell lysates following anti-MZF1 immunoprecipitation. **(H-I)** After transfection with MTSS1-AS overexpression plasmid or vector, PANC-1 cell lysates were immunoprecipitated with anti-MZF1 **(H)** or anti-STUB1 **(I)** antibody and then analyzed by Western blot analysis using anti-MZF1 and anti-STUB1. **(J)** Lysates of PANC-1 cells transfected with the MTSS1-AS overexpression plasmid or/and siSTUB1 were immunoprecipitated with anti-MZF1 before analysis by Western blot analysis using anti-MZF1 or anti-ubiquitin antibodies. **(K)** MZF1, STUB1 and MTSS1 proteins in PANC-1 cells transfected with siSTUB1 and/or the MTSS1-AS overexpression plasmid and cultured under acidic or normal conditions were measured by Western blot analysis. All data were presented as means ± SD of at least three independent experiments. Values are significant at $^{**}P < 0.01$ as indicated. NS means the difference is not significant.

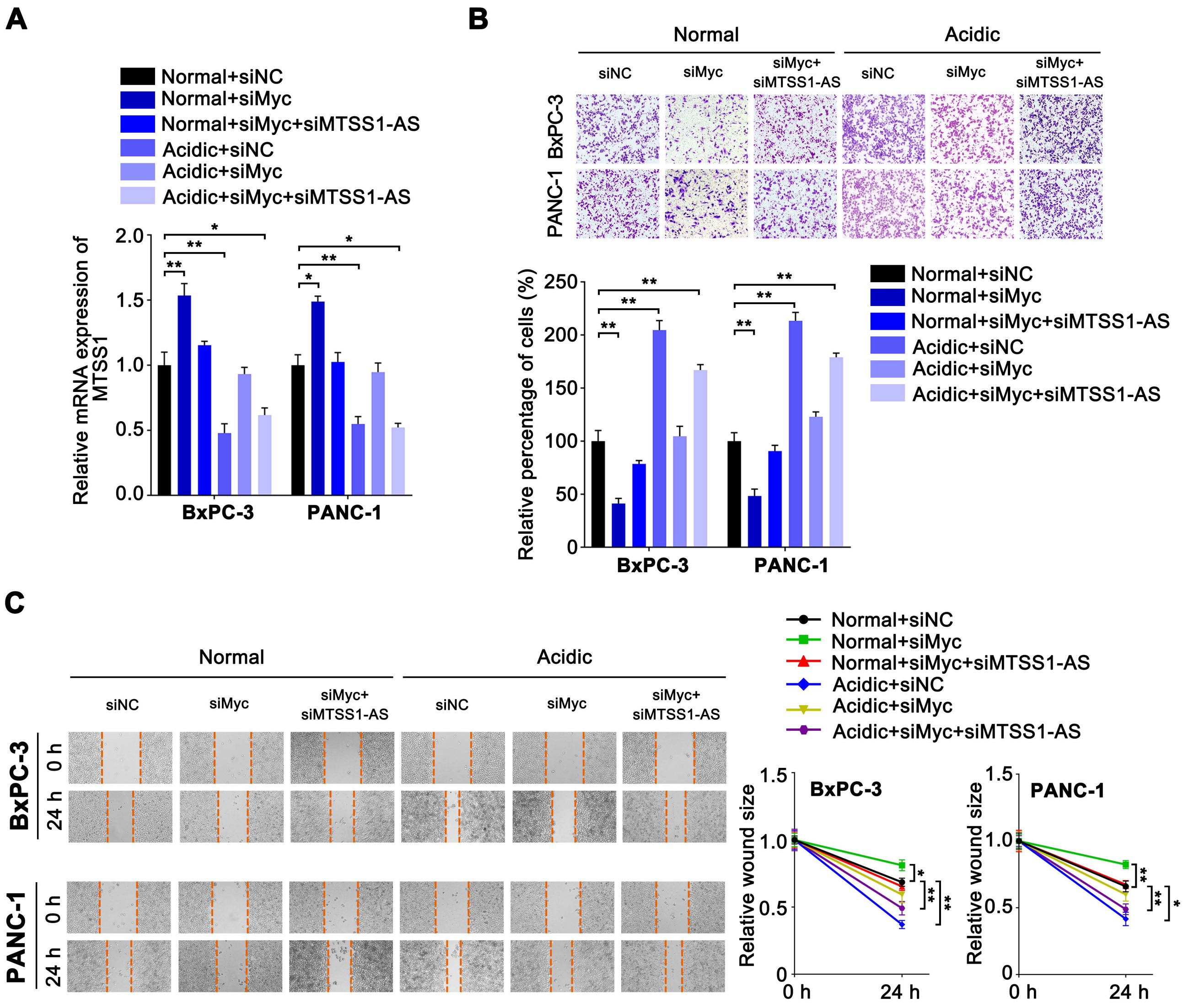

A
Normal+siNC
Normal+siMyc
Normal+siMyc+siMTSS1-AS
Acidic+siNC
Acidic+siMyc
Acidic+siMyc+siMTSS1-AS
Relative mRNA expression of MTSS1
BxPC-3
PANC-1
B
Normal
Acidic
siNC
siMyc
siMyc+ siMTSS1-AS
BxPC-3
PANC-1
Relative percentage of cells (%)
C
Normal
Acidic
siNC
siMyc
siMyc+ siMTSS1-AS
BxPC-3
PANC-1
0 h
24 h
Relative wound size

Figure S5. Extracellular acidity decreased the expression of MTSS1-AS through Myc-mediated transcription inhibition. (A) MTSS1 mRNA levels were assessed by qRT-PCR in BxPC-3/PANC-1 cells transfected with siNC, siMyc or/and siMTSS1-AS and cultured in normal or acidic medium. **(B-C)** After BxPC-3/PANC-1 cells were transfected with siNC, siMyc or/and siMTSS1-AS and cultured under normal or acidic conditions, the invasion **(B)** and migration **(C)** ability were detected by Transwell and wound healing assays. **(B)** Representative images (Upper) and relative percentage of invaded cells (Below) are shown. **(C)** Representative images (Left) and relative wound size (Right) of two time points (0 h, 24 h) in the six groups are shown. All data were presented as means ± SD of at least three independent experiments. Values are significant at $*P < 0.05$, $**P < 0.01$ as indicated.
